# Influence of the rearing system on the ileum microbiome, metabolome, and transcriptome in meat rabbits

**DOI:** 10.3389/fvets.2024.1456790

**Published:** 2024-11-08

**Authors:** Zhoulin Wu, Xiaoyu Li, Maoqin Xu, Bin Wen, Xiangchao Fu, Zhonghua Tang, Xueqin Liu, Jiamin Zhang

**Affiliations:** ^1^Meat Processing Key Laboratory of Sichuan Province, College of Food and Biological Engineering, Chengdu University, Chengdu, China; ^2^Sichuan Academy of Grassland Science, Chengdu, China; ^3^Sichuan Aichi Rabbit Food Co., Ltd., Bazhong, China

**Keywords:** rabbit, rearing system, ileal microbiome, metabolome, ileal mucosa transcriptome

## Abstract

The rearing system of livestock plays a vital role in animal production, meat quality, and overall welfare. This study aimed to assess the influence of cage-rearing system and forest-rearing system on the ileum microbiota, metabolome, and ileal mucosa transcriptome in meat rabbits. Moreover, 16S rDNA sequencing revealed significant differences in the ileal microbiome composition: caged rabbits exhibited a higher abundance of the genera uncultured *Erysipelotrichaceae* and *Delftia*, whereas the levels of *Muribaculaceae*, unclassified *Burkholderiales*, and uncultured *Eubacteriaceae* were lower compared to rabbits reared in the forest. Metabolome analysis identified 372 differentially accumulated metabolites in the ileum content, which were predominantly mapped to amino acid metabolism, nucleotide metabolism, and energy metabolism pathways. The cage-rearing system was found to positively correlate with the efficient utilization of nutrient sources. Additionally, transcriptome analysis of the ileal mucosa revealed 984 differentially expressed genes, predominantly involved in metabolic pathways, signal transduction pathways, and immune response processes. Through Pearson correlation analysis, we were able to elucidate the metabolic pathway, immune responses, and disease resistance mechanisms were affected by the rearing system. Overall, the findings suggested that metabolic adaptation, nutrient utilization, and immune response play crucial roles in how rabbits adjust to different rearing systems. While the cage system may enhance nutrient efficiency, it appears to suppress immune function and disease resistance.

## 1 Introduction

Rabbit meat consumption and production are not widespread globally, but they have developed into a highly specialized livestock industry in some Asian countries and most Mediterranean countries, particularly in China, Italy, France, and Spain (1). Among these countries, China alone accounts for approximately 60% of the world's total production, with Europe being the second-largest producer (1, 2). Rabbits are considered ideal for meat production due to their many advantageous qualities, such as a short vital cycle and gestation period, significant daily weight gain, and high fertility. As highly specialized monogastric herbivores, rabbits possess a digestive system well-adapted to a high-fiber diet, enabling them to have remarkable feed conversion efficiency.

Rabbit meat is also lean, rich in essential amino acids, and contains highly unsaturated fats (3). It provides moderate energy levels and low cholesterol content (1). Despite these beneficial qualities, rabbit meat consumption is declining in Western countries, largely due to concerns over animal welfare and consumer preferences (4).

In recent years, there has been growing consumer interest in animal welfare, organic farming, meat nutrition and human health. Many consumers prefer to buy meat products from outdoor rearing systems due to their superior sensor qualities compared to those from conventional housing systems (5, 6). For rabbits, the rearing system is one of the factors that moderately affects growth performance, behavior, immunity, oxidative stress, and carcass and meat quality (7–9). For instance, outdoor rearing systems are associated with the expression of more natural behaviors and a lower incidence of digestive disorders (10). Digestive disorders are a major cause of welfare impairment, with a high occurrence rate in conventional housing systems.

The ileum, the terminal part of the small intestine, harbors trillions of microbes that intimately interact with the host (11). The composition of the resident microbiome is influenced by the host's physiological condition and, in turn, impacts overall health (12). Some studies have shown that the intestinal microbiota can directly interact with intestinal epithelial cells and further modulate the intestinal immune system (13), epithelium differentiation (14), and immune system-mediated mucosal protection (15).

Currently, the effects of rearing systems on intestinal histomorphology and gut microbial composition have been extensively explored in pigs (18), chickens (19), and geese (20). Similarly, previous studies conducted on rabbits have concluded that different rearing systems significantly affect growth performance, slaughter yield, and meat composition (21) while also reducing the incidence of digestive disorders (10). Importantly, integrative analysis of the microbiome, transcriptome, and metabolome provides novel insight into how host-microbiota interactions affect animal performance and their overall welfare (16, 17). To date, the complex interactions between intestinal microbiota and host genetic responses in meat rabbits reared under different rearing systems remain largely unexplored.

Therefore, to address this knowledge gap, a multi-omics approach was employed to explore the ileal bacterial composition, metabolome, and host gene expression in meat rabbits reared either in cages or in a forest environment. This approach aims to identify key microbiota, uncover regulatory metabolic pathways, and clarify the molecular mechanisms underlying physiological responses. Ultimately, these results are expected to identify host-microbe associations and provide a comprehensive view of the biological systems involved, offering valuable insights into how rearing systems contribute to the welfare of rabbit farming.

## 2 Materials and methods

### 2.1 Experiment design and animal treatment

A total of 30 healthy male New Zealand rabbits from a purebred line were used in this study. From 18 to 20 days of age, young animals were gradually introduced to solid feed, alongside breast milk, to help them adapt to pelleted food. At 30 days of age, all rabbits were weaned and provided with commercial pelleted food (the diet ingredients are shown in Supplementary Table 1), fed *ad libitum* three times daily at 8:00, 13:00, and 18:00, respectively.

At 40 days of age, the rabbits were randomly assigned to two groups based on their rearing systems: cage-rearing system (RC) and forest-rearing system (RF). The RC group was individually housed in stainless steel cages with a density of 0.2 m^2^/head under standard conditions with temperatures between 15°C and 23°C. In the RF system, a forest area of approximately 200 m^2^ was enclosed by a 3-m high metal fence, with a calculated stocking density of 13 m^2^/head. Both groups were fed the same commercial pelleted food three times daily *ad libitum*, and water was freely available through valve self-drinkers throughout the 50-day experimental period.

### 2.2 Sample preparation

At 90 days of age, six rabbits with similar body weights (2,180.5 ± 102.5 g) from each group were randomly selected. The selected animals were subjected to electrical stunning, followed by exsanguination, skinning, and evisceration procedures. Immediately after slaughter, ileum content samples were collected aseptically, snap-frozen in liquid nitrogen, and stored at −80°C for subsequent microbial and metabolomic analyses. At the same time, sections of the ileum were collected, and the digesta was washed away from the epithelial lining using ice-cold sterile phosphate-buffered saline (PBS). Subsequently, the ileum mucosa was gently scraped after being washed three times with PBS and then quickly stored in liquid nitrogen for RNA sequencing.

### 2.3 Analysis of ileum content-associated microbiota by *16S rRNA* gene sequencing

The bacterial DNA extraction, amplification, library construction, and sequencing were conducted as previously described (22). Briefly, frozen ileum content samples were subjected to microbial genomic DNA extraction using the QIAamp DNA Stool Mini Kit (Qiagen, Shanghai, China) according to the manufacturer's protocol. The DNA concentration and purity were evaluated using a NanoDrop ND-1000 spectrophotometer (NanoDrop Technologies, Montchanin, DE, USA). The V3–V4 hypervariable region of the bacterial *16S rRNA* gene was amplified by PCR using specific primers 338F and 806R. After PCR amplification, all qualified amplicons were further subjected to library construction and subsequently sequenced on the Illumina HiSeq 2500 platform, generating 250-bp paired-end reads.

Raw reads were filtered and analyzed using QIIME2 software (23). Tags were clustered into operational taxonomic units (OTUs), and then, the taxonomic assignment was conducted using the SILVA v138 database (silva-138–99-nb-classifier.qza) with the classify-sklearn algorithm. Alpha and beta diversities were calculated using the Kruskal–Wallis test and the PERMANOVA method, respectively (23, 24). Statistical analyses were conducted using R software (v4.1.3) (https://www.r-project.org/). The criterion of significance was determined at a *P*-value of <0.05, and the values were presented as means. Finally, PICRUST2 (v1.7.3) (25) was utilized to predict the functional profiles of the *16S rRNA* gene data, and pathways were predicted using the KEGG database.

### 2.4 Analysis of ileum content-associated metabolomics by LC-MS

Liquid chromatography-mass spectrometry (LC-MS/MS) technology was used to analyze the metabolic profiling of intestinal content, following a previously described method (26). Briefly, metabolites were extracted using a 400 μL methanol solution (4:1, v/v) with 0.02 mg/mL L-2-chlorophenylalanin as an internal standard. The mixture was sonicated at 40 kHz for 30 min at 5°C, followed by protein precipitation at −20°C for 30 min. Subsequently, the supernatant was obtained by centrifugation at 13,000 rpm for 10 min at 4°C. Finally, the supernatant was evaporated to dryness under a gentle stream of nitrogen for LC-MS analysis, and a pooled quality control sample (QC) was prepared by mixing equal amounts of metabolites from each sample to ensure data consistency.

The LC-MS analysis was conducted using the UHPLC-Q Exactive HF-X system from Thermo Fisher Scientific, following conditions outlined in our previous study (27). For liquid chromatographic separation, a flow rate of 0.25 mL min^−1^ was maintained, and the column temperature was set to 40°C. Each sample was equilibrated, and a 2 μL volume was injected for analysis. Mass spectral data were acquired using spray voltages of 3.8 kV for positive ion mode (ESI+) and −2.5 kV for negative ion mode (ESI^−^).

Finally, the raw LC-MS data were processed using Progenesis QI software (Waters Inc., Milford, MA, USA). Metabolite identification was conducted by searching the reference standard MS/MS spectral libraries or databases such as the HumanMetabolome Database (HMDB, http://www.hmdb.ca), Metlin (http://metlin.scripps.edu), and mzCloud (https://www.mzcloud.org) database. Differentially accumulated metabolites (DAMs) were identified based on a variable importance in projection (VIP) threshold >1.0 in the OPLS-DA model and a *p*-value of <0.05 in a student's *t*-test. Functional enrichment analysis of the DAMs was conducted using the Kyoto Encyclopedia of Genes and Genomes (KEGG) database.

### 2.5 Analysis of the ileal mucosa transcriptome profiling by RNA sequencing

Total RNA was extracted from the ileal mucosa using TRIzol Reagent (TaKaRa, Dalian, China), and DNA was removed using the RNeasy Midi Kit (Qiagen, Valencia, CA, USA) according to the manufacturer's protocols. RNA quality was assessed with a 5,300 Bioanalyser (Agilent Technologies, USA) and quantified using the NanoDrop ND-2000 spectrophotometer (NanoDrop Technologies).

The RNA-seq transcriptome library was constructed using the NEBNext Ultra^TM^ RNA Library Prep Kit of Illumina (NEB, USA) according to the manufacturer's instructions. The library sequencing was conducted on the Illumina NovaSeq™ X Plus platform, generating 150 bp paired-end reads.

The raw reads were subjected to adaptor removal and quality control, with low-quality reads filtered as described in our previous report (28). The cleaned data were then mapped to the latest rabbit reference genome (*OryCun*2.0.110 in Ensembl) using HISAT2 software (v2.2.1) with default parameters (29). Gene expression was quantified using featureCounts (v2.0.1) (30), which counted the number of mapped reads for each gene. Differentially expressed genes (DEGs) between the two groups were analyzed using the DESeq2 R package (v3.2.3) (31), with significant DEGs defined by an adjusted *P*-value (Padj) of <0.05 and a |log2 (FoldChange)| of >1.

Finally, the DAVID (v6.8) (32) software was used to analyze the statistical enrichment of Gene Ontology (GO) terms and Kyoto Encyclopedia of Genes and Genomes (KEGG) pathways, with an FDR threshold of <0.05 indicating significance.

### 2.6 Statistical analysis

All results from six replicates per group are presented as mean value ± standard deviation (SD). An unpaired Student's *t*-test was used to compare the two groups using SPSS 21.0 (IBM Corp., New York, USA), with statistical significance defined at a *P*-value of <0.05. The Spearman's correlation coefficient and cluster analysis were conducted using the R package (v4.2.0), and all results were visualized using ggplot2 (v3.3.6) in the R package (33). Pearson's correlation coefficient was used to identify significant correlations in the multi-omics data, with *P*-values of <0.05 considered statistically significant.

## 3 Results

### 3.1 Rearing system induced a shift in the ileum content microbiota composition

**Figure 1:**
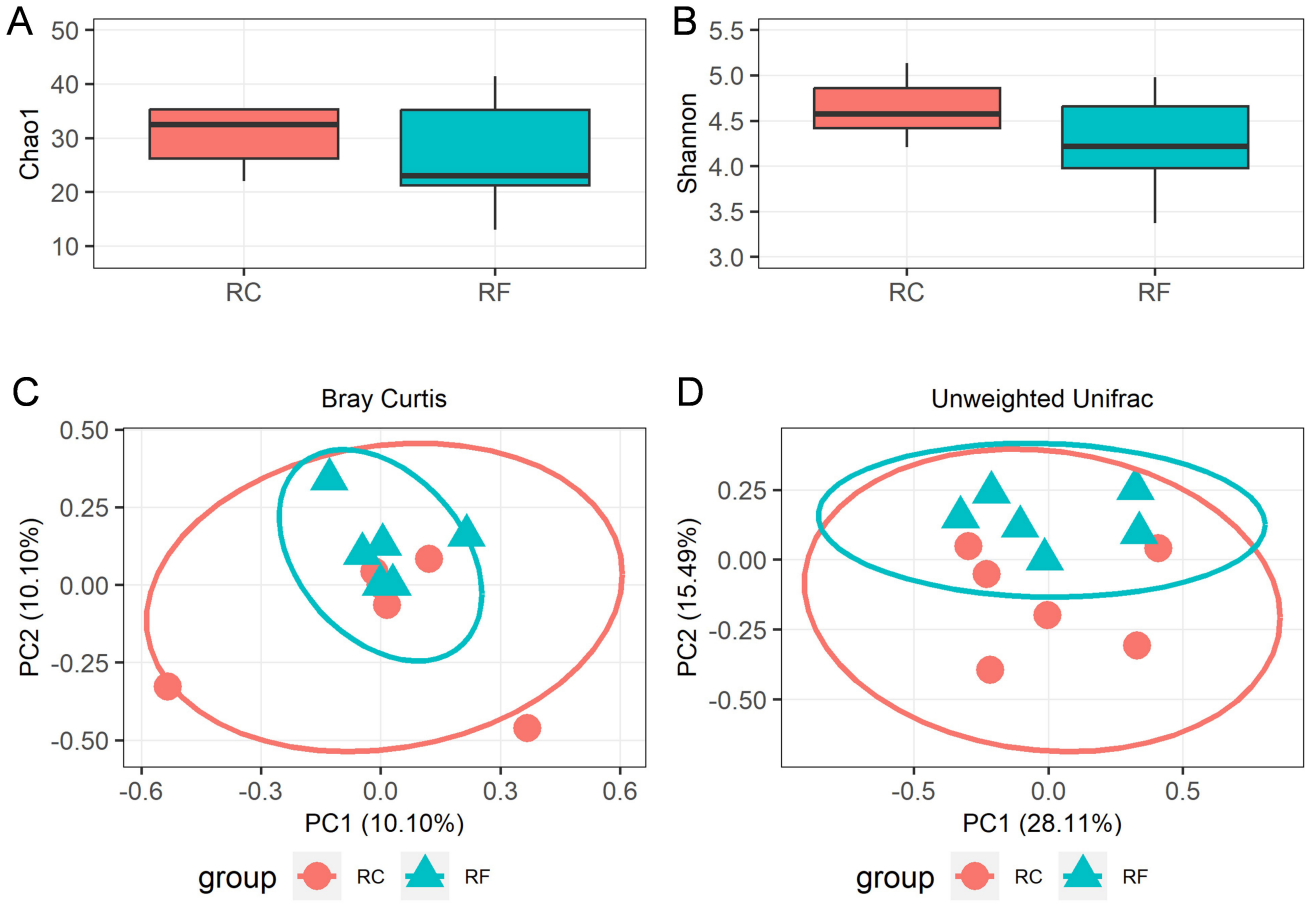
Alpha diversity and beta diversity indices of ileal content microorganisms in RF and RC. Comparison of the richness (Chao1) **(A)** and diversity (Shannon) **(B)**. The overall microbiota structures were shown by principal coordinate analysis (PCoA) of Bray-Curtis distances **(C)** and unweighted UniFrac distances **(D)**. RF, forest rearing system; RC, cage rearing system.

A total of 1,119,688 bacterial sequences with an average length of 448 bp were retained and categorized into 396 operational taxonomic units (OTUs) using the DEBLUR program. There were no significant differences in alpha diversity indices between the two rabbit groups, as indicated by Chao1 richness (Figure 1A) and the Shannon diversity (Figure 1B) index (*P* > 0.05). However, a notable shift in beta diversities was observed through principal coordinates analysis (PCoA) based on Bray–Curtis and unweighted unifrac methods. PCoA plots revealed that the ileum content microbiota of RF animals clustered together and were clearly separated from those of RC rabbits (Figures 1C, D), indicating that the bacterial communities were positively correlated with the rearing system.

**Figure 2:**
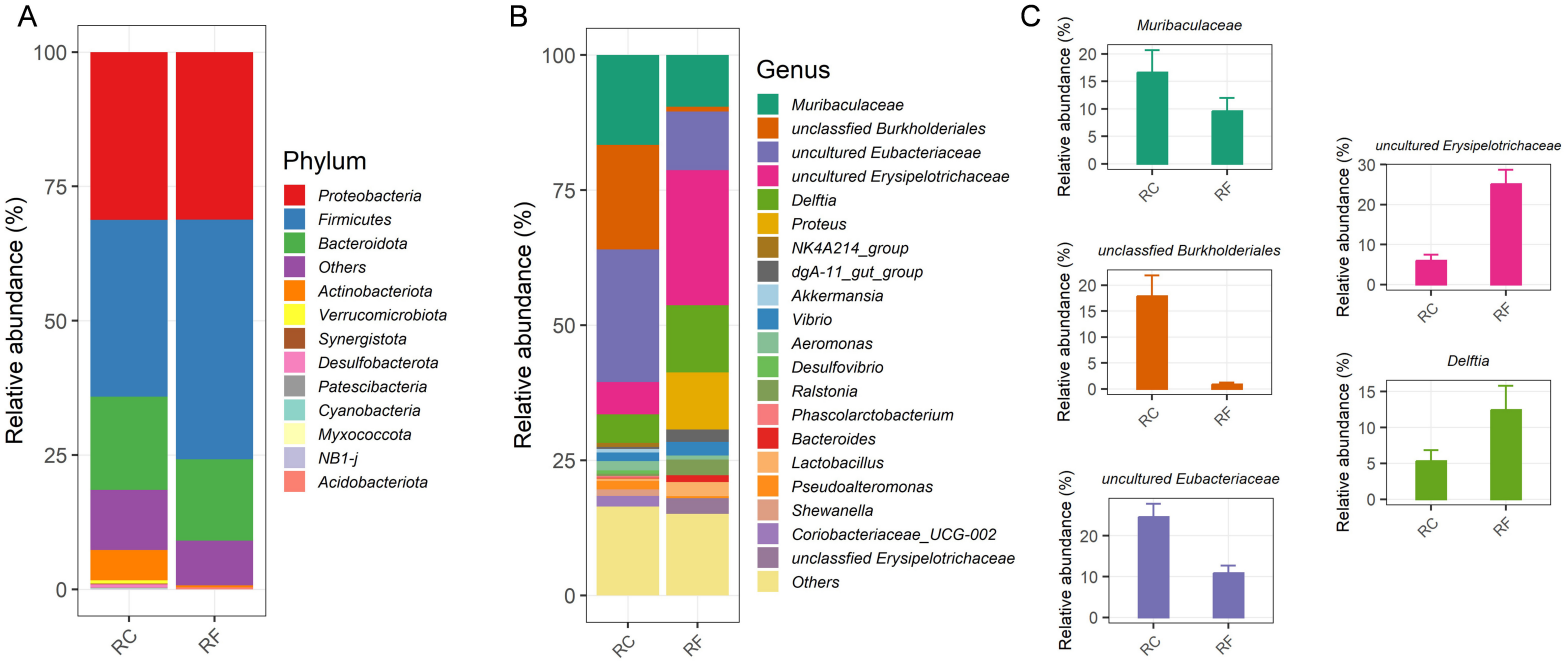
Different rearing systems altered the specific ileal bacterial compositions of rabbits. Relative abundance of the ileal phylum taxonomic level **(A)**. Relative abundance of the top 20 bacteria at the genus taxonomic level **(B)**. Most changed bacteria genera **(C)**. RF, forest rearing system; RC, cage rearing system.

At the phylum level, the ileum content microbiota was predominantly composed of four major phyla in both rabbit groups: *Proteobacteria, Firmicutes, Bacteroidota*, and *Actinobacteriota*, which together accounted for 89.24% of the OTUs (Figure 2A).

Significant differences were observed between the groups in the relative abundance of *Firmicutes* and *Actinobacteriota*, with *Firmicutes* being more abundant in RF rabbits and *Actinobacteriota* more prevalent in RC rabbits (Figure 2A). The average bacterial community compositions of the top 20 genera are shown in Figure 2B. Furthermore, significant differences were noted in the abundance of the five most prevalent genera between RF and RC rabbits. RF rabbits exhibited increased colonization of several genera, including uncultured *Erysipelotrichaceae, Delftia*, and *Proteus*. However, the RC rabbits had higher abundances of *Muribaculaceae*, unclassified *Burkholderiales*, and uncultured *Eubacteriaceae* in their ileum content (Figure 2C).

### 3.2 Predicted functions of the ileum content microbiota

**Figure 3:**
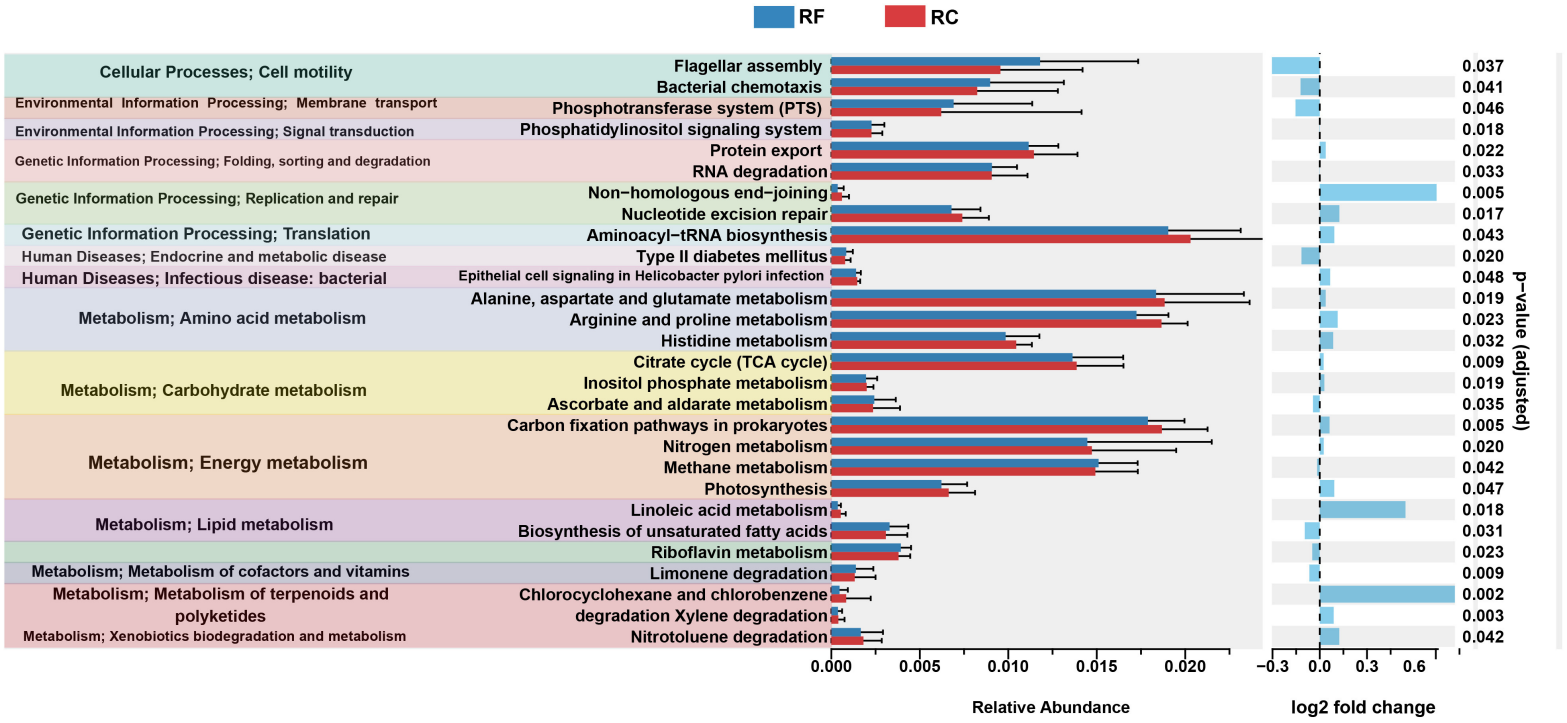
Significantly differing ileal microbiota pathways between RF and RC rabbits. All OTUs were used to predict their functions against the KEGG database through PICRUST2 software (v1.7.3). Difference values are presented as the difference from RF to RC group. RF, forest rearing system; RC, cage rearing system.

Using PICRUST2 analysis, the functional profile of the ileum content microbiota was inferred based on the rearing system, revealing 28 significantly enriched KEGG pathways between the two groups. In rabbits reared in the forest system, pathways related to lipid metabolism, such as the biosynthesis of unsaturated fatty acids, riboflavin metabolism, and limonene degradation, were highly represented (Figure 3).

In addition, pathways associated with cellular processes, including flagellar assembly and bacterial chemotaxis, as well as pathways involved in the phosphotransferase system, type II diabetes mellitus, and ascorbate and aldarate metabolism, were more prominent in RF rabbits (Figure 3).

In contrast, rabbits in the cage-rearing system showed higher expression of pathways related to amino acid metabolism, including histidine, alanine, aspartate, glutamate, arginine, and proline metabolism. Energy metabolism pathways, such as carbon fixation in prokaryotes, nitrogen metabolism, and photosynthesis, were also more pronounced in the RC rabbits compared to the RF group (Figure 3).

### 3.3 The rearing system induced a shift in the ileum content metabolomic profile

**Figure 4:**
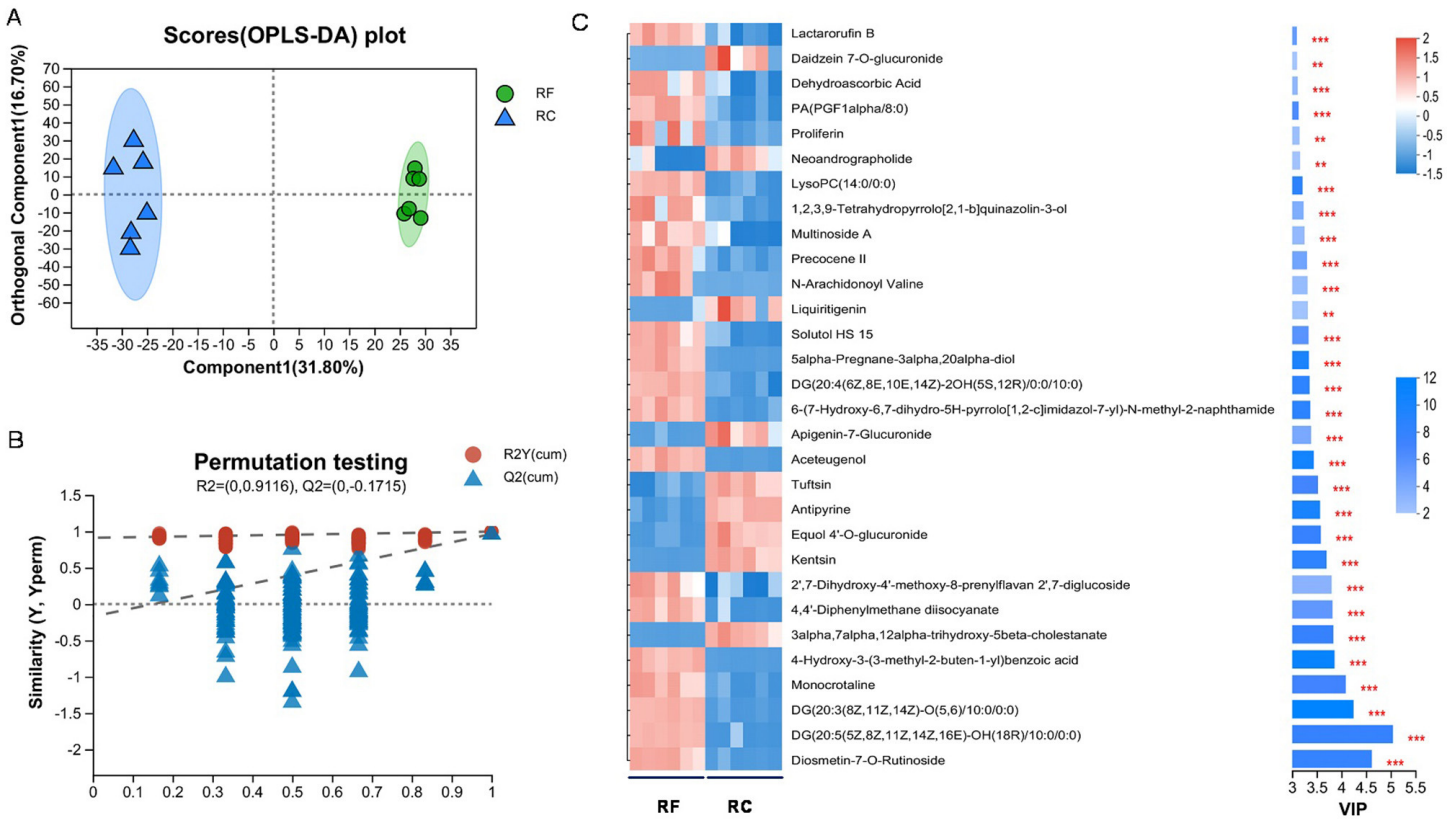
Metabolome analysis of ileum content samples from different rearing systems. Orthogonal partial least squares discriminant analysis (OPLS-DA) score plots **(A)** and permutation tests were obtained for RF and RC groups **(B)**. Top 30 differentially accumulated metabolites in the ileum content of rabbits identified by OPLS-DA **(C)** (*0.01 <*P* ≤ 0.05; **0.001 <*P* ≤ 0.01; ***P ≤ 0.001). RF, forest rearing system; RC, cage rearing system.

A total of 1,174 metabolites were detected in the ileum content, with 627 identified in positive ion mode and 547 in negative ion mode. The OPLS-DA analysis indicated a clear separation between the two rabbit groups (Figures 4A, B), indicating distinct metabolic profiles between them with stable and reliable models. Based on the thresholds of a VIP of > 1.00 and *P*-value of <0.05, 372 DAMs were identified, of which 181 were upregulated and 191 were downregulated (Supplementary Table 2).

These DAMs were classified into 13 categories, such as 118 lipids and lipid-like molecules, 59 organic acids and derivatives, 47 organoheterocyclic compounds, 44 phenylpropanoids and polyketides, 27 organic oxygen compounds, and 22 benzenoids, among others. Notably, lipids and lipid-like molecules, organic acids and derivatives, organoheterocyclic compounds, and phenylpropanoids and polyketides accounted for 33.62%, 16.81%, 13.39%, and 12.54% of the DAMs, respectively (Supplementary Figure 1). Then, a cluster heatmap analysis of the top 30 metabolites further confirmed that RF and RC rabbits could be distinctly separated based on their metabolomic profiles (Figure 4C).

**Figure 5:**
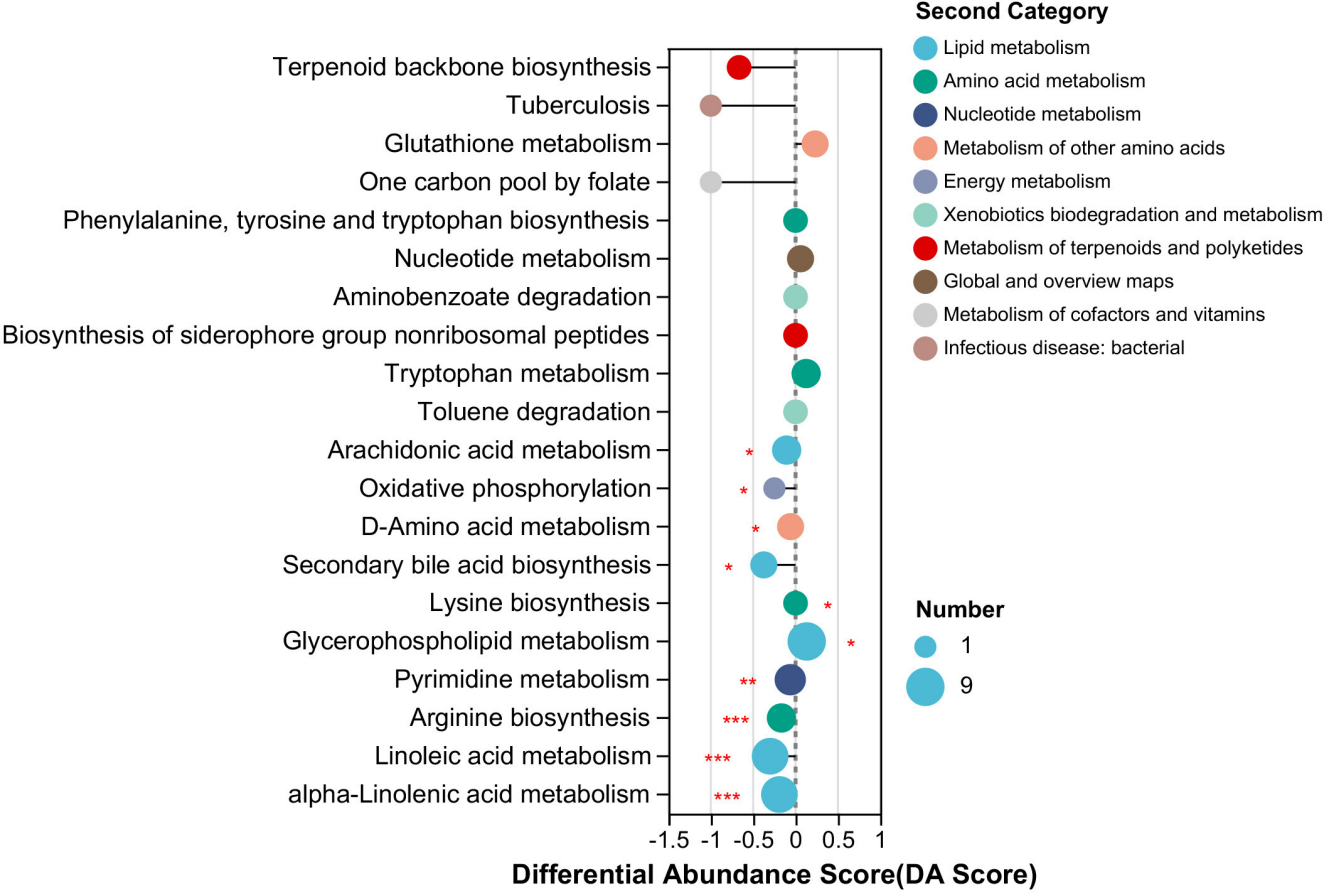
Significant differing ileal metabolomic pathways based on differentially accumulated metabolites from RF and RC rabbits. The length of the line segment represents the absolute value of the differential abundance score, and the size of the dots refers to the number of metabolites in the pathway (*0.01 <*P* ≤ 0.05; **0.001 <*P* ≤ 0.01; ***P ≤ 0.001). RF, forest rearing system; RC, cage rearing system.

The KEGG pathway enrichment analysis of DAMs from RF and RC rabbits showed that the DAMs were primarily enriched in 10 significant KEGG pathways, among which eight pathways were downregulated and two pathways were upregulated in RF rabbits compared to RC rabbits. The enriched pathways included lipid metabolism, amino acid metabolism, energy metabolism, nucleotide metabolism, and other amino acid metabolism pathways (Figure 5). Notably, the majority of the altered metabolites exhibited a higher abundance in RC rabbits, with the majority being involved in lipid metabolism pathways, such as alpha-linolenic acid metabolism, linoleic acid metabolism, secondary bile acid biosynthesis, and arachidonic acid metabolism (Figure 5).

### 3.4 Gene expression profile of ileal mucosa under different rearing systems

**Figure 6:**
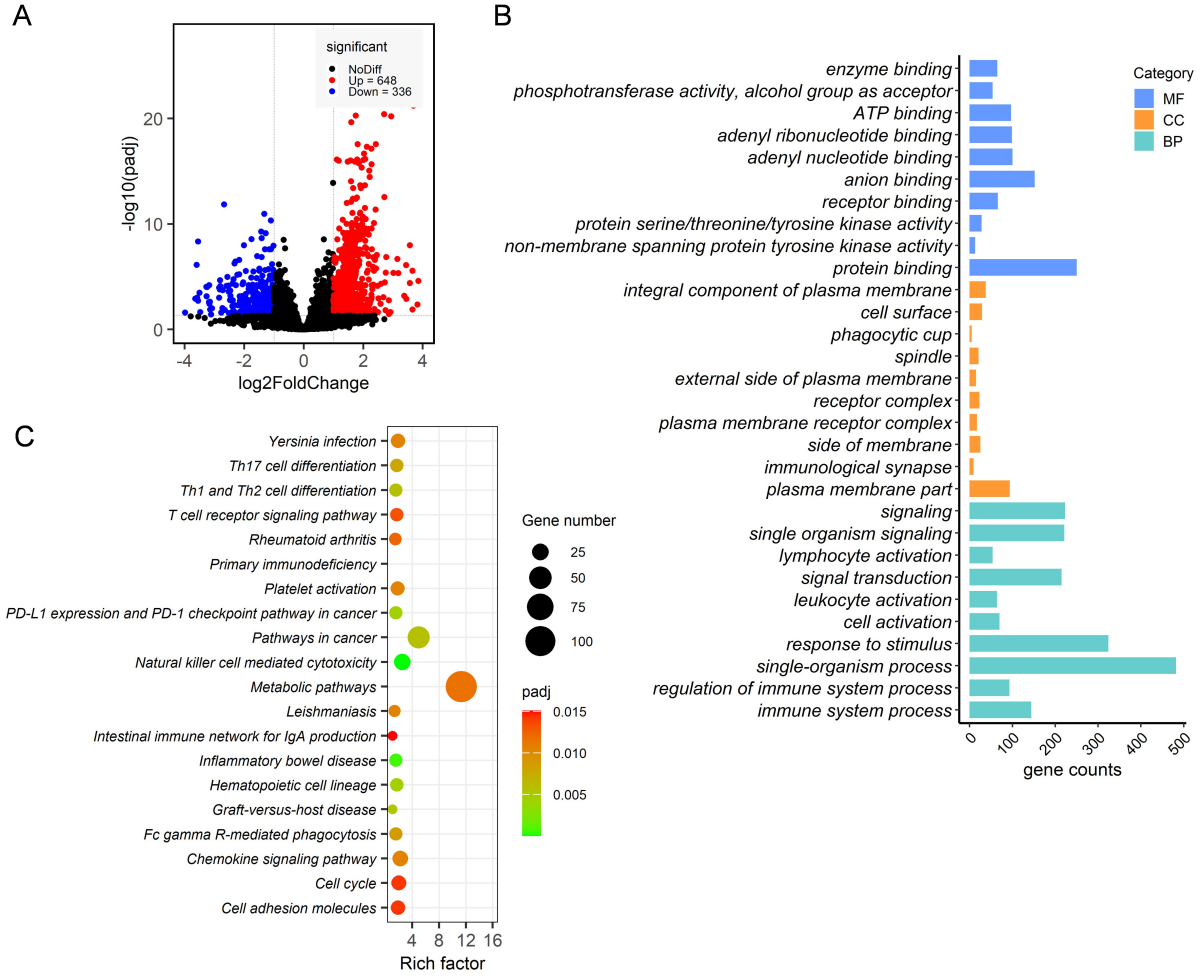
Transcriptome analysis of ileal mucosa samples from RF and RC rabbits. Volcano plot of differentially expressed genes **(A)**. The top 10 highly significant GO terms in each group include MF, CC, and BP **(B)**. Significantly enriched KEGG pathways are potentially related to metabolism, disease resistance and the immune response process **(C)**. RF, forest rearing system; RC, cage rearing system.

A total of 567,304,736 raw reads were generated from 12 ileum epithelial samples. After filtering adaptor sequences and low-quality reads, a total of 562,212,578 clean reads of 150 base pairs were retained. Over 82.72% of these clean reads were successfully mapped to the rabbit genome using HISAT2 software. After filtering genes with no more than 10 raw count reads in at most two samples, a total of 9,734 annotated genes were identified, representing 46.05% of the 21,140 gene set. Overall, 984 DEGs were detected in the ileum epithelia, with 648 DEGs upregulated and 336 DEGs downregulated in RF rabbits compared to the RC group (Figure 6A). These DEGs were defined using a threshold of |log2(FoldChange)| > 1 and Padj of <0.05.

GO and KEGG pathway enrichment pathways were conducted to assess the biological processes and pathways associated with these DEGs. The GO enrichment analysis revealed that the DEGs were enriched in 335 GO terms, with the 10 most significant terms summarized for each category.

In the biological process (BP) category, the DEGs were primarily associated with cell activation, immune system processes, and their regulation. In the molecular function (MF) category, the DEGs were majorly associated with kinase activity and binding. For the cellular component (CC) category, the DEGs were mainly enriched in processes related to signaling at the immunological synapse, receptor complexes, phagocytic cup formation, and the plasma membrane (Figure 6B). KEGG pathway analysis showed that DEGs were significantly enriched in metabolic pathways, signal transduction pathways, including the chemokine signaling pathway and the T-cell receptor signaling pathway, and immune response processes, such as graft-vs.-host disease, Th1 and Th2 cell differentiation, Th17 cell differentiation, Fc gamma R-mediated phagocytosis, and primary immunodeficiency (Figure 6C).

### 3.5 Correlation analysis of ileum bacteria, metabolites and host gene expression

The influence of the rearing system on the three omics-microflora, metabolome, and host gene expression—was explored. For metabolome features, 78 annotated differentially accumulated metabolites (DAMs) enriched in lipid, amino acid, and energy metabolism were included.

**Figure 7:**
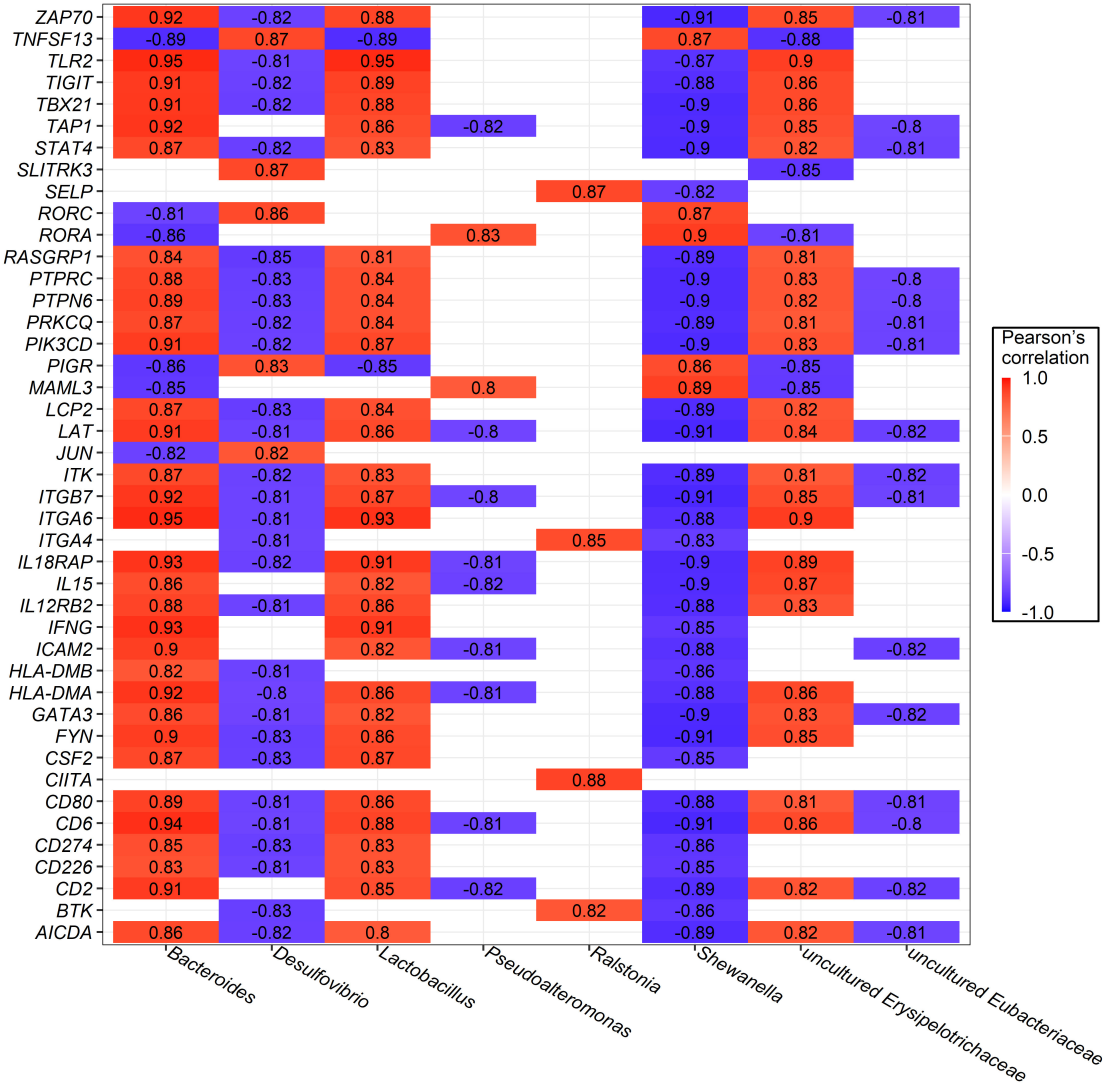
Significant Pearson's correlations between ileum bacterial genera and ileal mucosa genes involved in immune response and metabolic pathway. Red represents a positive correlation, while blue represents a negative correlation.

Additionally, the top 10 most abundant bacterial genera and a subset of 43 DEGs enriched in immune response processes and metabolic pathways were selected for Pearson's correlation analysis. Only coefficients with |r| > 0.8 and *P-*values of <0.05 are shown in Figure 7. Significant correlations were found between bacteria and host gene expression, with *Bacteroides* strongly and positively correlated with 32 genes, *Lactobacillus* with 31 genes, and uncultured *Erysipelotrichaceae* with 26 genes. In contrast, unclassified *Burkholderiales* were negatively correlated with 36 genes, while *Shewanella*, and *Desulfovibrio* were negatively correlated with 35 and 29 genes, respectively.

Furthermore, the relationships between bacterial communities and metabolites provided insights into the functions of key microbiota and metabolites under different rearing systems. Correlations with coefficients |r| > 0.8 and *P*-values of <0.05 are shown in Supplementary Figure 2. Among them, *Bacteroides* showed a broad range of strong and significant correlations, with 25 positive and 28 negative correlations with metabolites. In addition, uncultured *Erysipelotrichaceae* was positively correlated with 12 metabolites and negatively correlated with 8 metabolites. Interestingly, we also observed that the genera *Lactobacillus* was positively correlated with 14 metabolites, while uncultured *Eubacteriaceae* was positively correlated with 12 metabolites.

Taken together, these results indicate that specific bacteria, specifically *Bacteroides*, uncultured *Erysipelotrichaceae*, and *Lactobacillus*, might play important roles in interacting with numerous metabolites and host genes, influencing the adaptation of the rearing system by modulating host immune responses and metabolic pathways.

## 4 Discussion

To meet the growing consumer demand for sustainable purchase choices, previous studies have examined the effects of rearing systems on the growth performance and welfare of livestock. In particular, the gut microbiome is a crucial determinant of animal health and productivity, and its composition is well known to be associated with the rearing environment. For example, Lin et al. (34) demonstrated that geese reared indoors exhibited a higher abundance of pathogenic genera and lower levels of commensal genera compared to those raised outdoors.

Similarly, for broiler chickens, floor-reared birds showed a relatively higher abundance of potentially pathogenic and litter-associated bacteria (35), which could be due to increased exposure to environmental microbes. This exposure may enhance immune function and disease resistance (35, 36). Despite these insights into poultry, there remains a gap in understanding the impact of rearing systems on the gut microbiota of rabbits and the potential links between bacterial genera, host gene expression patterns, and the metabolome. Our 16S rDNA sequencing results revealed that the dominant phyla in both forest-reared (RF) and cage-reared (RC) rabbits were *Proteobacteria, Firmicutes, Bacteroidota*, and *Actinobacteriota*. Although relative abundances differed, these dominant phyla align with previous studies on the cecal microbiota of New Zealand White rabbits (37).

While overall bacterial communities were mostly similar, key taxa differences may reflect adaptations to different rearing environments. *Muribaculaceae*, unclassified *Burkholderiales*, and uncultured *Eubacteriaceae* were reduced, while uncultured *Erysipelotrichaceae* and *Delftia* were significantly increased in RF rabbits compared to RC rabbits. *Muribaculaceae* is considered beneficial and linked to pathways involving cytokines and short-chain fatty acids (15), with its abundance varying seasonally in rabbits (38). *Burkholderiales* include bacteria with diverse metabolic functions (39), while uncultured *Eubacterium*, a member of *Eubacteriaceae*, can produce butyrate, which plays a critical role in energy homeostasis, colonic motility, immunomodulation, and suppression of inflammation in the gut (40, 41).

The bacterial taxa *Erysipelotrichaceae* have been recurrently associated with dyslipidemic phenotypes in hosts, including mice and humans, particularly in the context of obesity, metabolic syndrome, and hypercholesterolemia (42). Furthermore, PICRUST2 analysis of these key taxa indicated that pathways related to lipid metabolism and disease resistance, such as the phosphotransferase system and type II diabetes mellitus, were more prominent in forest-reared (RF) rabbits. In contrast, functions associated with amino acid and energy metabolism were more prevalent in cage-reared (RC) rabbits. These findings suggest that the shifts in gut microbiota composition may have led to substantial changes in host metabolism and disease resistance, potentially explaining why cage-reared rabbits demonstrated favorable growth performance (21). This is also consistent with previous findings on rabbits (20, 43) and broiler chickens (35) reared in cage systems.

The intestine is not only the primary digestive organ but also an important immune organ in animals. It plays a major role in the digestion and absorption of nutrients from ingested food, while the intestinal mucosa functions as a key component of the physical and chemical barriers, as it can recognize and combat pathogen infections, maintaining homeostasis between the host and the commensal gut microflora (44). Numerous studies have demonstrated the influence of gut microbiota composition on host intestinal epithelium gene expression and intestinal mucosal immune function (45, 46).

In the present study, we explored the effects of different rearing systems on gene expression in the ileal mucosa using RNA-seq analysis. We identified a total of 984 DEGs between rabbits reared in the RF and those in the RC group. These genes were found to be implicated in multiple biological processes and pathways, with many involved in immune system processes and their regulation. Moreover, KEGG enrichment analysis revealed that these DEGs were significantly enriched in metabolic pathways, signal transduction pathways, and immune response processes, such as Th1 and Th2 cell differentiation, Th17 cell differentiation, Fc gamma R-mediated phagocytosis, and primary immunodeficiency. Previous studies have reported that compared to caged chickens, ground-floor-reared birds exhibited higher levels of IL-1β and IFN-γ mRNA in the ileum (47).

Similarly, our transcriptome and metagenome results indicated that rabbits reared in the forest exhibited stronger intestinal mucosal immune function. Notably, immune-related genes such as *IL9, IL15, IL2RG, IL12RB2, IL1RN, IL18RA, IF2A, IRF1, IFNG, TLR2*, and *TLR8* were significantly upregulated in RF rabbits compared to those reared in cages. In line with our findings, Inman et al. (48) reported that piglets raised in an isolator had significantly increased *IL-2* levels produced by mucosal T cells and significantly reduced *IL-4* levels compared to piglets raised outdoors, further supporting the notion that rearing conditions can impact the immune response. These results suggest that changes in rearing conditions can lead to enhanced immune responses in the rabbit ileal mucosa at the transcriptome level.

The intestinal content serves as a valuable indicator of gut microbial activity and host metabolism. To investigate the metabolic response to changes in the rearing system, we compared the ileal content metabolome between RF and RC rabbits. The differentially accumulated metabolites between the two groups were primarily associated with lipid metabolism, amino acid metabolism, energy metabolism, and nucleotide metabolism (Figure 5). Notably, these pathways were more abundant in the cage-reared rabbits, suggesting a positive correlation between the cage-rearing system and the efficient utilization of nutrient sources and growth performance. This finding aligns with previous studies demonstrating that animals raised in floor conditions tend to have higher feed efficiency and superior growth performance across various livestock species, including ducks (49), chickens (47), and pigs (50).

Furthermore, a comprehensive correlation analysis across metagenomics, metabolomics, and transcriptomics revealed that specific bacterial genera, such as *Bacteroides, Lactobacillus*, and uncultured *Erysipelotrichaceae*, were found to be significantly positively associated with multiple metabolites involved in nutritional metabolism, as well as genes associated with immune response and metabolic pathways. These findings suggest that these bacteria may play an essential role in interacting with ileal metabolites and mucosa genes, thereby influencing the host's adaptation to different rearing systems by modulating immune responses and metabolic processes.

## 5 Conclusion

The present study demonstrated that the rearing system has a significant impact on the microbial composition, metabolomics of ileal content, and host transcriptomics. Rabbits reared in the forest exhibited the gut microbiome with a lower relative abundance of *Muribaculaceae*, unclassified *Burkholderiales*, and uncultured *Eubacteriaceae*, but a higher relative abundance of uncultured *Erysipelotrichaceae* and *Delftia* compared to those reared in cages.

In addition, the metabolomic profile of ileal content differed significantly between the groups, with changes primarily in pathways related to amino acid metabolism, nucleotide metabolism, and energy metabolism. Notably, the cage-rearing system was positively associated with improved nutrient utilization. However, significant transcriptional changes were also observed in the ileal mucosa, particularly in metabolic pathways, signal transduction, and immune response processes. Overall, while the cage-rearing system enhances nutrient utilization, it appears to be associated with a depressed immune response and reduced disease resistance.

## Data Availability

The RNA-seq data generated in this project are deposited in the Sequence Read Archive (SRA) repository, accession number PRJNA1105390. The 16S rRNA gene sequencing data are deposited in the SRA repository, accession number PRJNA1105432.
