# Supplementary material for: Influence of the rearing system on the ileum microbiome, metabolome, and transcriptome in meat rabbits
Source: Front Vet Sci. 2024 Nov 8;11:1456790. doi: 10.3389/fvets.2024.1456790 (PMC11582050; doi:10.3389/fvets.2024.1456790)
Supplement: Supplementary file 1 [file Data_Sheet_1.pdf]

Supplementary Material

1 Supplementary Figures and Tables

1.1 Supplementary Figures

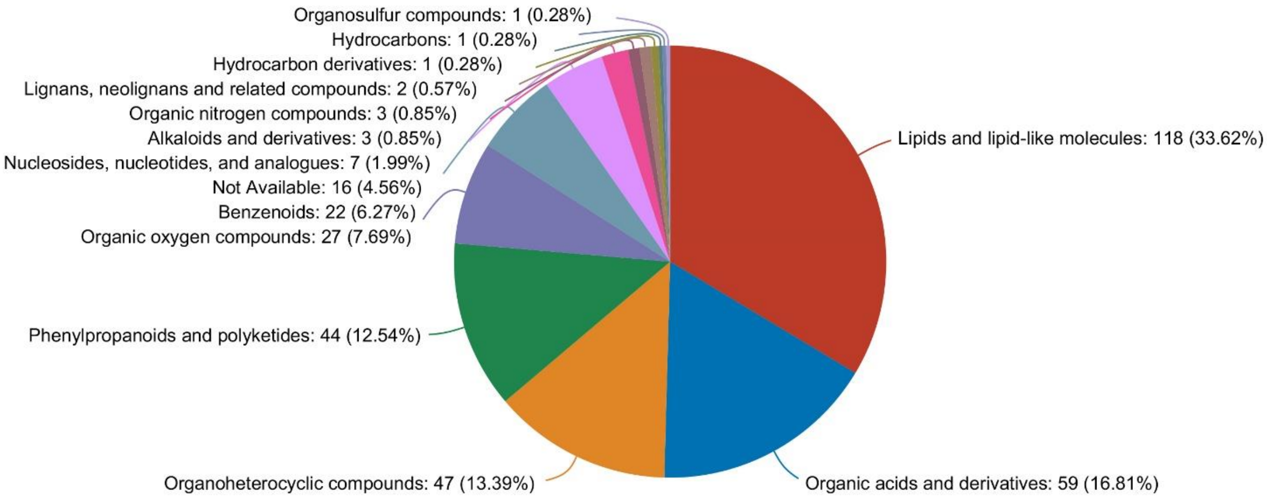

Supplementary Figure 1. Annotation of differentially accumulated metabolites in HMDB

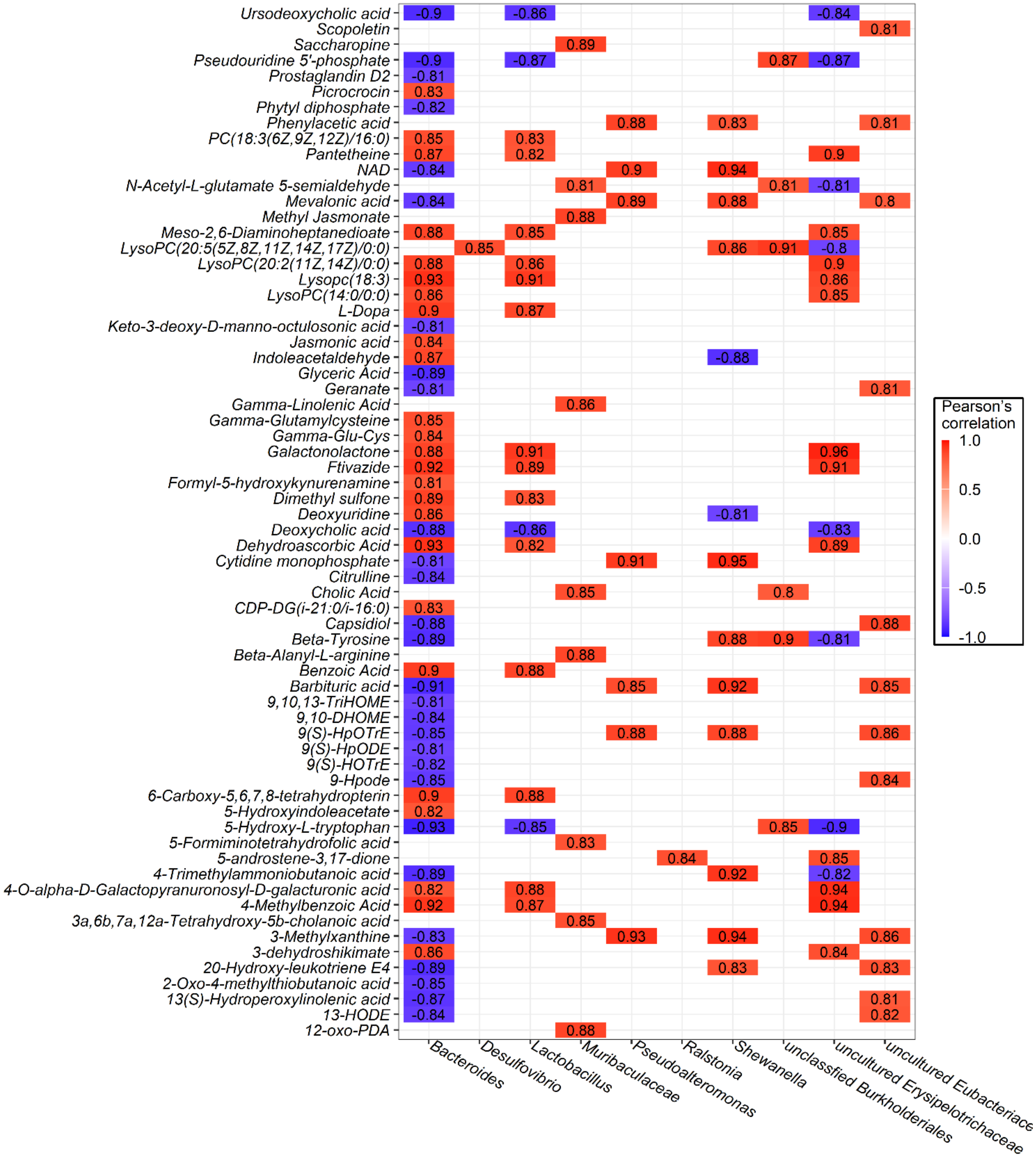

Supplementary Figure 2. Significant Pearson's correlations between ileum bacterial genera and ileum differentially accumulated metabolites. Red represents a positive correlation

1.2 Supplementary Tables

Supplementary Table 1. Ingredients and chemical component of the experimental diet.

**Ingredient**	**Diet (%)**
alfalfa grass	30.00
Barley grain	23.50
corn	8.50
wheat bran	10.00
soya bean meal	23.50
Molasses	0.35
choline chloride	0.15
Di-calcium phosphate	1.65
Sodium chloride	0.30
calcium hydrophosphate	0.35
DL-Methionine	0.20
Meat rabbit premix	1.50
Total	100
**Chemical Component**	
Dry matter (DM)	85.83
Crude protein (CP)	16
Crude fiber (CF)	14.25
Ether extract (EE)	3.51
Metabolizable energy (ME, MJ/Kg)	10.8MJ/kg
Calcium	0.9
Phosphorus	0.61
Lysine	0.91
Methionine	0.36
Threonine	0.59
Tryptophan	0.26

Supplementary Table 2. Differentially accumulated metabolites identified between RF and RC rabbits.

ID	Metabolite	Regulate	Mode	CAS ID	M/Z	Retention time	FC(RF/RC)	P_value	FDR
neg_2354	DG(20:5(5Z,8Z,11Z,14Z,16E)-OH(18R)/10:0/0:0)	up	neg	-	583.3457	6.745617	4.9211	1.15E-08	1.67E-06
neg_3732	Diosmetin-7-O-Rutinoside	up	neg	520-27-4	607.1701	5.177867	3.4734	8.18E-09	1.38E-06
pos_327	2',7-Dihydroxy-4'-methoxy-8-prenylflavan 2',7-diglucoside	up	pos	-	697.3054	5.238867	2.7016	0.000164	0.00541
neg_3201	DG(20:3(8Z,11Z,14Z)-O(5,6)/10:0/0:0)	up	neg	-	585.3614	6.666083	2.0015	1.20E-12	1.68E-09
pos_324	Antipyrine	down	pos	60-80-0	189.1016	5.332033	0.531	1.08E-10	8.43E-08
neg_1928	Monocrotaline	up	neg	315-22-0	695.2975	5.244783	2.2426	4.64E-08	4.77E-06
pos_1371	Aceteugenol	up	pos	93-28-7	207.1008	5.467617	1.9646	3.78E-11	3.94E-08
neg_3517	4-Hydroxy-3-(3-methyl-2-buten-1-yl)benzoic acid	up	neg	-	205.087	6.0984	3.3643	5.65E-12	4.76E-09
neg_3407	3alpha,7alpha,12alpha-trihydroxy-5beta-cholestanate	down	neg	547-98-8	471.3134	6.23495	0.4141	9.90E-09	1.55E-06
pos_1635	N-Arachidonoyl Valine	up	pos	-	426.2965	6.423883	3.049	0.000673	0.0136
pos_2592	Solutol HS 15	up	pos	61909-81-7	389.2649	6.72695	1.7893	7.80E-07	9.05E-05
neg_3897	4,4'-Diphenylmethane diisocyanate	up	neg	-	287.0239	3.648917	3.4733	1.53E-06	8.70E-05
pos_2800	LysoPC(14:0/0:0)	up	pos	20559-16-4	490.2913	6.307183	1.7057	2.39E-09	1.25E-06
neg_2847	Kentsin	down	neg	56767-30-7	535.2757	7.7107	0.4609	2.16E-09	5.06E-07
pos_3202	1,2,3,9-Tetrahydropyrrolo[2,1-b]quinazolin-3-ol	up	pos	-	206.1281	4.099783	2.0745	5.29E-05	0.002631
neg_3665	Equol 4'-O-glucuronide	down	neg	-	399.1101	5.70455	0.5038	1.09E-08	1.64E-06
pos_3242	Cenisertib	down	pos	-	493.2794	3.625217	0.6228	0.00013	0.004981
neg_3046	Tuftsin	down	neg	112592-90-2	535.2756	7.159367	0.565	8.24E-08	7.40E-06
pos_2629	MG(0:0/22:5(7Z,10Z,13Z,16Z,19Z)/0:0)	down	pos	-	427.2807	6.625983	0.5243	0.007242	0.05416

ID	Metabolite	Regulate	Mode	CAS ID	M/Z	Retention time	FC(RF/RC)	P_value	FDR
pos_323	Diosmin	up	pos	520-27-4	609.1792	5.174833	1.5998	8.55E-05	0.003771
pos_2696	20-Hydroxypregn-4-en-3-one	up	pos	145-14-2	349.2725	6.501683	1.5421	2.46E-08	7.69E-06
pos_1741	Pentadecanedioylcarnitine	down	pos	-	457.3282	6.773583	0.5645	0.000184	0.005662
neg_3712	Apigenin-7-Glucuronide	down	neg	-	427.0687	5.348067	0.523	2.57E-05	0.000809
neg_3654	Liquiritigenin	down	neg	578-86-9	255.0669	5.770617	0.162	0.002128	0.01994
pos_999	Ftivazide	up	pos	54-85-3	310.058	1.710083	1.474	1.50E-05	0.000922
neg_1869	6-(7-Hydroxy-6,7-dihydro-5H-pyrrolo[1,2-c]imidazol-7-yl)-N-methyl-2-naphthamide	up	neg	-	342.1028	4.671567	1.8969	1.34E-09	4.03E-07
neg_2303	DG(20:4(6Z,8E,10E,14Z)-2OH(5S,12R)/0:0/10:0)	up	neg	-	601.3562	6.610267	1.5175	2.43E-09	5.14E-07
neg_2348	5alpha-Pregnane-3alpha,20alpha-diol	up	neg	566-58-5	365.2708	6.73775	1.6624	1.28E-10	5.39E-08
pos_3144	(R)-Canadine	up	pos	2086-96-6	679.2949	5.0966	1.9615	0.005903	0.04765
neg_1939	Precocene II	up	neg	644-06-4	265.1089	5.366017	2.1751	1.14E-05	0.000418
pos_440	Calcidiol	down	pos	19356-17-3	423.3221	6.835567	0.656	0.002505	0.02967
neg_3761	Multinoside A	up	neg	59262-54-3	609.1498	4.91185	1.7782	0.000482	0.007787
neg_1881	Daidzein 7-O-glucuronide	down	neg	-	411.0739	4.79725	0.397	0.002737	0.02336
pos_3195	(2S,3S,4S,5R)-6-[4-(5,7-Dihydroxy-4-oxo-2,3-dihydrochromen-2-yl)phenoxy]-3,4,5-trihydroxyoxane-2-carboxylic acid	down	pos	-	431.0959	4.25265	0.551	0.001944	0.02619
pos_1462	Artemisin	up	pos	481-05-0	304.1566	5.9005	1.5105	3.27E-08	9.28E-06
neg_2028	Neoandrographolide	down	neg	-	525.2733	5.93145	0.4101	0.002122	0.01993
neg_3247	PA(PGF1alpha/8:0)	up	neg	-	617.3513	6.56245	1.5343	2.34E-07	1.79E-05
pos_1283	3-Hydroxy-cis-5-octenoylcarnitine	down	pos	-	302.1953	3.84135	0.5424	0.007505	0.05518
pos_3124	Papaverine	up	pos	58-74-2;61-25-6	679.2946	5.410433	1.5935	0.001333	0.02141
neg_4531	Dehydroascorbic Acid	up	neg	490-83-5	173.0088	0.976417	1.674	0.000315	0.005553
neg_3592	Quercetin 3,7-Dimethyl Ether	down	neg	2068/2/2	329.0678	5.97855	0.5731	6.79E-05	0.001799
neg_3171	Proliferin	up	neg	-	443.2822	6.7536	2.0115	0.001135	0.01352
pos_1197	5-hydroxyindole thiazolidine carboxylate	up	pos	-	279.0789	3.05455	1.4896	0.000946	0.01703
neg_2276	Carindone	up	neg	38045-62-4	533.2932	6.522567	1.3968	1.56E-07	1.26E-05

ID	Metabolite	Regulate	Mode	CAS ID	M/Z	Retention time	FC(RF/RC)	P_value	FDR
neg_598	Lactarorufin B	up	neg	-	281.1402	6.011733	1.6874	3.73E-06	0.000166
pos_2496	Ethyl icosapentate	up	pos	73310-10-8;86227-47-6	363.2879	6.990867	1.4125	1.33E-06	0.000119
pos_2787	7-Ketolithocholic Acid	down	pos	4651-67-6	408.3093	6.361683	0.6862	0.000202	0.005969
pos_4060	Beta-Alanyl-L-arginine	down	pos	-	309.1646	0.479483	0.6615	0.000478	0.01061
pos_320	HYPEROSIDE	up	pos	482-36-0	465.1013	5.05065	1.4531	0.000223	0.006141
neg_2027	Pravastatin	down	neg	81093-37-0	469.2402	5.93145	0.4911	0.002663	0.02323
pos_1656	PS(20:4(8Z,11Z,14Z,17Z)/20:1(11Z))	down	pos	-	870.5825	6.48615	0.7523	0.000539	0.01158
neg_3561	Difenoxin	up	neg	28782-42-5	445.1893	6.02725	1.6839	0.000102	0.0024
neg_3406	Astaxanthin	down	neg	472-61-7	631.3523	6.23495	0.63	1.88E-05	0.000638
neg_1216	Gamma-Glu-Cys	up	neg	636-58-8	249.0556	0.960617	1.9358	0.000968	0.01215
neg_3706	PA(22:6(4Z,7Z,11E,13Z,15E,19Z)-2OH(10S,17)/8:0)	up	neg	-	677.2866	5.397183	1.5135	6.37E-05	0.001724
pos_3646	Barbituric acid	down	pos	67-52-7	274.0772	1.124017	0.6679	0.001257	0.02029
neg_1970	3-ethylphenyl Sulfate	down	neg	-	201.0227	5.631433	0.5386	0.00022	0.004272
neg_599	Retrocalamin	up	neg	74729-98-9	443.1732	5.99665	1.5865	0.000121	0.002726
pos_3162	Hv-NCC-1	up	pos	135972-64-4	661.2845	4.830133	1.4521	0.001146	0.01941
neg_4276	3-Chloro-4-methyl-7-hydroxycoumarin	down	neg	-	244.9763	1.707567	0.614	3.46E-07	2.39E-05
neg_3826	Schaftoside	down	neg	207461-10-7;51938-32-0	563.1435	4.283467	0.6076	0.002069	0.01961
neg_3197	Dehydrocurdione	up	neg	38230-32-9	513.3243	6.682067	1.4455	6.99E-06	0.000273
pos_3270	12S-HHT	down	pos	54397-84-1	325.1747	3.381533	0.6719	0.003586	0.03556
neg_1626	Tetramethylscutellarein	up	neg	1168-42-9	323.0896	2.858417	1.6425	0.001273	0.01436

ID	Metabolite	Regulate	Mode	CAS ID	M/Z	Retention time	FC(RF/RC)	P_value	FDR
pos_2560	Monensin A	down	pos	17090-79-8	693.4162	6.7658	0.7568	0.003306	0.03375
neg_3111	DG(i-18:0/0:0/18:1(12Z)-O(9S,10R))	down	neg	-	635.529	6.936283	0.7384	6.84E-07	4.18E-05
neg_1984	Soyasaponin A1	down	neg	78693-94-4	633.2981	5.707633	0.645	0.000539	0.008353
pos_2930	Geldanamycin	down	pos	30562-34-6	602.3088	6.045067	0.772	0.001137	0.01936
pos_1425	Scopolamine	down	pos	51-34-3	321.1798	5.86105	0.7137	0.000303	0.007539
neg_3819	4-O-p-Coumaroylquinic acid	up	neg	-	337.0941	4.3987	1.5004	0.000109	0.002549
pos_934	Galactonolactone	up	pos	2426-46-2	223.0197	1.186333	1.4185	5.63E-05	0.00275
neg_1805	Cepharanthine	down	neg	481-49-2	651.271	3.992683	0.5495	0.006846	0.04264
pos_891	5-(4-Acetoxy-1-butynyl)-2,2'-bithiophene	up	pos	1219-28-9	299.0178	0.999317	1.3325	0.001068	0.01859
pos_2776	Avocadyne 4-acetate	down	pos	28884-46-0	344.2785	6.385033	0.7316	0.01061	0.06624
pos_3557	Milbemycin A4	down	pos	-	294.1537	1.694517	0.6892	0.007792	0.0561
neg_2108	Estrone glucuronide	up	neg	2479-90-5	427.1781	6.114417	1.4039	4.71E-06	0.000195
pos_3689	Gamma-Glutamylcysteine	up	pos	636-58-8	251.0687	0.983783	1.2731	0.000656	0.01334
pos_2972	Guan-fu base A	down	pos	-	471.2508	5.958417	0.7613	0.00018	0.005662
neg_683	5b-Cyprinol sulfate	down	neg	-	553.2865	7.415017	0.7013	0.007237	0.04423
neg_2216	Cholic Acid	down	neg	81-25-4	389.271	6.347	0.6936	0.009037	0.05185
neg_1801	Formyl-5-hydroxykynurenamine	up	neg	958733-17-0	461.1685	3.959017	1.5725	0.006719	0.04234
neg_1902	Dehydrovomifoliol	up	neg	15764-81-5	267.1245	5.039383	1.3623	0.000111	0.00256
neg_1760	3-Hydroxyphenylacetylglutamine sulfate	up	neg	-	368.9801	3.604567	1.4586	0.000228	0.004398
pos_2831	Prostaglandin D2	down	pos	41598-07-6	335.2182	6.23715	0.7783	0.001704	0.02483
neg_3476	Tetranor 12-HETE	up	neg	-	311.1871	6.1544	1.3708	5.77E-06	0.000232
pos_3129	Oleoside	down	pos	-	429.08	5.354283	0.6972	0.01951	0.09372
pos_2643	Gamma-Linolenic Acid	down	pos	506-26-3	311.2569	6.602733	0.8022	0.00253	0.02968
neg_1882	N-Acetyl-D-tryptophan	down	neg	2280/1/5	245.0937	4.80285	0.7179	0.000237	0.004474
neg_2215	Dihydrocapsiate	down	neg	-	307.1923	6.347	0.6949	0.000509	0.008116

ID	Metabolite	Regulate	Mode	CAS ID	M/Z	Retention time	FC(RF/RC)	P_value	FDR
neg_3834	2-Hydroxy-4,7-dimethoxy-2H-1,4-benzoxazin-3(4H)-one	up	neg	149182-67-2	246.0389	4.193633	1.4269	9.45E-05	0.002276
pos_845	Dimethyl sulfone	up	pos	67-71-0	95.01638	0.8672	1.295	0.000228	0.006141
pos_2565	MG(0:0/20:1(11Z)/0:0)	down	pos	-	429.2964	6.7658	0.8258	0.002772	0.03123
pos_3107	Stercobilin	down	pos	34217-90-8	595.3469	5.693083	0.7907	0.005568	0.04638
neg_3772	Naringenin 4'-O-glucuronide	down	neg	158196-35-1	429.0844	4.81705	0.6886	0.001562	0.01626
neg_2174	Capsidiol	down	neg	37208-05-2	281.1766	6.242917	0.7096	3.99E-05	0.001184
neg_3842	Agar	up	neg	9002-18-0	373.0876	4.095317	1.562	0.007154	0.04385
pos_3624	Azlocillin	up	pos	37091-66-0	500.0986	1.217533	1.2674	0.002897	0.03206
neg_493	Enterolactone	down	neg	185254-87-9;78473-71-9	297.1141	5.971017	0.6324	0.01383	0.06925
pos_418	Pregnanetriolone	down	pos	603-99-6	351.2498	6.602733	0.8011	0.01061	0.06624
neg_3856	Caffeic Acid	up	neg	4361-87-9;331-39-5;501-16-6	179.0347	3.971783	1.3089	0.000407	0.006776
pos_3370	5-(Methoxycarbonyl)thiophene-2-carboxylic acid	up	pos	-	204.0319	2.862567	1.2549	9.01E-06	0.000614
neg_3735	Cinncassiol C3	down	neg	64979-94-8	381.1933	5.1569	0.6685	0.0027	0.02323
neg_2229	PE(20:5/0:0)	up	neg	-	498.2647	6.370917	1.3146	0.000393	0.006621
pos_2678	MG(22:4(7Z,10Z,13Z,16Z)/0:0/0:0)	down	pos	-	371.2932	6.540533	0.8365	0.002715	0.03092
neg_4799	Moniliformin	up	neg	31876-38-7	142.998	0.665933	1.3092	9.07E-06	0.000338
neg_3759	Egtazic acid	up	neg	-	425.1405	4.924383	1.5153	0.006161	0.04035
neg_3276	PE(18:1(12Z)-2OH(9,10)/22:0)	down	neg	-	868.5761	6.490667	0.7739	0.01027	0.05649
neg_3850	L-Prolinamide, 5-oxo-L-prolyl-L-norvalyl-	up	neg	-	345.1567	4.03045	1.4854	0.002848	0.02416
pos_3547	Aurintricarboxylic acid	up	pos	-	445.0565	1.710083	1.2601	0.000653	0.01334

ID	Metabolite	Regulate	Mode	CAS ID	M/Z	Retention time	FC(RF/RC)	P_value	FDR
neg_1820	Sporol	down	neg	101401-88-1	301.1204	4.155233	0.6109	0.01314	0.06653
pos_50	Neocnidilide	down	pos	4567-33-3	195.1374	6.4317	0.8042	0.004678	0.04174
pos_2943	Dihydrokaempferol	down	pos	480-20-6	271.0591	6.01675	0.767	0.01009	0.06557
pos_3843	Saccharopine	down	pos	997-68-2	309.1645	0.657867	0.7714	0.01878	0.09162
pos_618	Melezitose	down	pos	597-12-6	527.1566	0.650067	0.7754	0.01496	0.08121
pos_1818	Bufadienolide	up	pos	29565-35-3	372.2919	7.153967	1.1934	0.006864	0.05308
pos_3632	Neobignonoside	up	pos	-	533.1087	1.170767	1.1914	0.000323	0.007913
pos_1626	9,12,13-TriHOME	down	pos	-	295.2259	6.392833	0.8475	0.000238	0.006254
neg_3610	5-Oxozaleplon	up	neg	-	320.115	5.95075	1.5511	0.007704	0.04634
pos_2583	Napelline	up	pos	5008-52-6	360.2556	6.734733	1.2623	0.004422	0.04038
neg_3542	Picrocrocin	up	neg	138-55-6	329.1616	6.057767	1.3649	0.000692	0.009892
pos_3612	Niflumic Acid	up	pos	4394-00-7	327.0303	1.317983	1.1702	0.00091	0.01665
pos_589	Avocadene 1-acetate	down	pos	24607-09-8	351.2507	6.392833	0.8348	0.004293	0.03978
neg_2152	5(6)-Epoxy Prostaglandin E1	up	neg	-	413.2193	6.203033	1.3524	6.92E-05	0.001806
pos_3035	Glycitein	down	pos	40957-83-3	285.0748	5.88825	0.753	0.01322	0.07557
neg_2280	Cirsimaritin	up	neg	6601-62-3	359.0785	6.538583	1.2794	2.07E-06	0.000113
neg_2165	Geranate	down	neg	459-80-3	379.2117	6.226983	0.7417	0.000222	0.004294
pos_2641	Carboprost	down	pos	35700-23-3	391.2436	6.602733	0.7792	0.02867	0.1198
neg_1715	(2S,3S,4S,5R)-3,4,5-Trihydroxy-6-[2-hydroxy-5-(3,5,7-trihydroxy-3,4-dihydro-2H-chromen-2-yl)phenoxy]oxane-2-carboxylic acid	up	neg	-	465.1057	3.289033	1.3061	0.004556	0.03324
pos_567	Stearidonic Acid	down	pos	20290-75-9	277.2153	6.501683	0.8533	0.002107	0.02742
pos_1700	Arbaprostil	down	pos	-	349.2338	6.633767	0.8256	0.01471	0.08069
pos_3318	Pantetheine	up	pos	496-65-1	261.1258	3.05455	1.2594	0.003206	0.03322

ID	Metabolite	Regulate	Mode	CAS ID	M/Z	Retention time	FC(RF/RC)	P_value	FDR
neg_2058	Genistein	down	neg	529-59-9;446-72-0	269.0462	6.004667	0.8141	0.000289	0.005182
pos_370	Corchorifatty acid F	down	pos	95341-44-9	293.2101	6.0527	0.8245	0.005761	0.04725
neg_3846	3-Methoxyphenol sulfate	up	neg	-	203.0019	4.059967	1.427	0.000489	0.007868
pos_1262	Hippuric Acid	up	pos	495-69-2	180.0649	3.593317	1.1692	0.000211	0.006085
neg_3642	Norethindrone	down	neg	68-22-4	319.1674	5.85315	0.7692	0.002325	0.02109
neg_3912	Chlorogenic Acid	up	neg	202650-88-2;327-97-9;202650-88-2	353.0891	3.543067	1.302	0.001381	0.015
pos_3643	6-Hydroxybenzothiazole	down	pos	-	184.0421	1.139633	0.7522	0.009388	0.06337
neg_3756	(3S,5R,6R,7E)-3,5,6-Trihydroxy-7-megastigmen-9-one	up	neg	-	287.1509	4.9994	1.4444	0.01224	0.06365
neg_1173	1-(3-Carboxypropylcarbamoyl)-5-fluorouracil	up	neg	-	294.0299	0.8808	1.2088	1.15E-07	9.87E-06
neg_1771	Labetalol	up	neg	36894-69-6	365.1286	3.7059	1.5228	0.01959	0.0858
pos_208	NAD	down	pos	53-84-9;58-68-4	664.1138	1.030467	0.8432	0.001556	0.02367
neg_3478	3a,6b,7a,12a-Tetrahydroxy-5b-cholanoic acid	down	neg	80875-93-0	405.2662	6.1464	0.734	0.01984	0.08626
pos_1166	Phenylacetic acid	down	pos	103-82-2	311.0686	2.925083	0.757	0.0157	0.08289
pos_2716	Trenbolone	up	pos	10161-33-8	288.1982	6.462767	1.1579	2.15E-06	0.000182
neg_3752	Isoquercitrin	up	neg	21637-25-2;482-35-9	463.0903	5.0348	1.2163	0.000188	0.003811
pos_3868	(R)-1-Methylpiperidine-2-carboxylic acid	down	pos	-	144.1016	0.6424	0.8781	1.67E-07	3.50E-05
neg_3156	DG(16:0/0:0/20:3(6,8,11)-OH(5))	down	neg	-	633.5138	6.785383	0.8395	3.09E-06	0.000151
pos_1263	Benzoic Acid	up	pos	55-21-0;303-07-1;65-85-	105.0336	3.5973	1.1899	0.000284	0.007177

ID	Metabolite	Regulate	Mode	CAS ID	M/Z	Retention time	FC(RF/RC)	P_value	FDR
				0;99-10-5;100-52-7					
pos_3216	L-Felinine	up	pos	-	190.089	3.918133	1.1622	0.000132	0.004995
pos_640	(2r,3r,4s,5r,6s)-2-(Hydroxymethyl)-6-Sulfanyl-Oxane-3,4,5-Triol	up	pos	-	238.0735	0.88265	1.1723	7.25E-06	0.000504
pos_960	QUISQUALIC ACID	down	pos	52809-07-1	231.0716	1.4786	0.8466	0.00106	0.01854
neg_1892	Baicalin	down	neg	21967-41-9	445.0796	4.89405	0.7608	0.005681	0.03869
pos_203	Creatinine aspartate	down	pos	-	260.098	1.061633	0.8263	0.004291	0.03978
pos_3208	12-Hydroxydodecanoic acid	down	pos	505-95-3	258.2055	4.04605	0.8089	0.004754	0.04205
pos_3749	Lactobionic acid	up	pos	-	403.0825	0.843883	1.1182	1.01E-06	9.33E-05
neg_1016	D-Apiose	down	neg	639-97-4	149.045	0.650083	0.8343	0.000254	0.004726
pos_405	Bempedoic acid	down	pos	-	309.2414	6.509383	0.8496	0.01596	0.08374
neg_4827	Tezacitabine	up	neg	-	294.0298	0.650083	1.2215	2.76E-07	2.04E-05
neg_3426	(11E)-Octadec-11-enedioylcarnitine	down	neg	-	492.2757	6.211033	0.7697	0.00218	0.02025
neg_2307	9(S)-HpOTrE	down	neg	111004-08-1	309.2081	6.61825	0.8244	0.000818	0.01123
neg_3906	6-Carboxy-5,6,7,8-tetrahydropterin	up	neg	-	246.0389	3.590883	1.2173	0.000164	0.00342
pos_288	1,2,3,4-Tetrahydroisoquinoline-1-carboxylic acid	up	pos	-	178.0857	3.249517	1.1914	0.002135	0.02751
pos_2872	Prostaglandin I2	down	pos	35121-78-9	353.229	6.15945	0.8443	0.007856	0.05644
pos_2567	Estrone	up	pos	481-97-0;53-16-7	288.1983	6.7658	1.1823	0.006586	0.05169
pos_1575	[(S)-1-Carboxy-2-phenyl-ethyl]-carbamoyl-Arg-Val-Arg-aldehyde	down	pos	-	604.3439	6.167233	0.8202	0.01051	0.06624
pos_3693	N-Acetyl-L-glutamate 5-semialdehyde	down	pos	13074-21-0	156.065	0.975983	0.8245	0.00165	0.02468
neg_2232	Lithocholic acid glycine conjugate	down	neg	474-74-8	432.3135	6.3789	0.8213	0.01504	0.07325
neg_3760	Rubiadin	down	neg	117-02-2	253.0511	4.915217	0.7061	0.02332	0.09659
pos_419	LysoPC(22:5(7Z,10Z,13Z,16Z,19Z)/0:0)	up	pos	-	570.3535	6.602733	1.1538	0.002193	0.02781
pos_3157	1-(2,2-Difluoroethyl)pyrrolidine-3,4-dicarboxylic acid	up	pos	-	224.0732	4.888267	1.2014	0.002527	0.02968

ID	Metabolite	Regulate	Mode	CAS ID	M/Z	Retention time	FC(RF/RC)	P_value	FDR
pos_1321	LysoPE(18:4(6Z,9Z,12Z,15Z)/0:0)	down	pos	-	438.2373	4.47485	0.8243	0.0399	0.1457
neg_3829	Chrysin-7-O-Glucuronide	down	neg	-	429.0844	4.250783	0.7964	0.00427	0.03187
neg_3376	DG(10:0/PGJ2/0:0)	up	neg	-	583.3664	6.2912	1.2164	0.001548	0.01618
pos_82	12-oxo-PDA	down	pos	85551-10-6	293.21	6.37725	0.8612	0.01328	0.0757
neg_3410	9(S)-HpODE	down	neg	29774-12-7	311.2237	6.23495	0.8675	0.000106	0.002481
pos_1597	N-Acetylsphinganine	down	pos	-	344.3148	6.23715	0.8195	0.007347	0.05466
neg_3325	3,4-Dimethyl-5-pentyl-2-furanpropanoic acid	up	neg	116627-39-5	283.1557	6.3869	1.3593	0.0119	0.06248
neg_3316	Phytyl diphosphate	down	neg	-	491.2128	6.394917	0.8228	0.000171	0.003509
neg_583	8-Hydroxycarteolol	up	neg	-	923.5099	6.203033	1.2005	3.67E-06	0.000166
neg_3804	N1,N10-Dicoumaroylspermidine	down	neg	65715-79-9	436.2259	4.4983	0.7989	0.01433	0.07084
neg_2312	(9S,10S)-9,10-dihydroxyoctadecanoate	down	neg	-	315.2549	6.626233	0.8342	0.000989	0.01224
pos_908	Hyaluronan biosynthesis, precursor 1	down	pos	-	344.0978	1.053833	0.7792	0.01572	0.08289
pos_3203	CDP-DG(i-21:0/i-16:0)	up	pos	-	534.7834	4.078817	1.1827	0.01274	0.07357
neg_2219	Beta-Thujaplicin	up	neg	499-44-5	491.2446	6.347	1.2019	0.000307	0.00547
pos_1011	Tranexamic Acid	down	pos	1197-18-8	190.1432	1.73155	0.781	0.03466	0.135
neg_4278	3-Methylglutaric Acid	down	neg	-	145.05	1.707567	0.8586	3.06E-05	0.000957
pos_446	N-Stearoyl Cysteine	up	pos	-	370.2764	6.936533	1.1276	0.006275	0.0504
pos_3723	4-O-alpha-D-Galactopyranuronosyl-D-galacturonic acid	up	pos	5894-59-7	415.0459	0.9215	1.2407	0.02288	0.104
neg_3762	Indolelactic acid	down	neg	7417-65-4	204.0666	4.907267	0.7804	0.003723	0.02907
pos_899	N4-Acetylcytidine	down	pos	3768-18-1	324.0592	1.022667	0.8313	0.01374	0.07726
neg_3749	Astilbin	up	neg	29838-67-3	449.1109	5.0562	1.2948	0.007456	0.04524
neg_4518	Threoninyl-Tryptophan	down	neg	186761-42-2	342.0855	0.99235	0.7703	0.01223	0.06365
pos_584	Methylenecyclohexane	down	pos	-	215.1787	6.40055	0.8662	0.004917	0.04278

ID	Metabolite	Regulate	Mode	CAS ID	M/Z	Retention time	FC(RF/RC)	P_value	FDR
neg_3611	Dihydrogenistein	down	neg	21554-71-2	271.0619	5.95075	0.7588	0.02724	0.1047
pos_2947	1-Undecanol	down	pos	112-42-5	383.3259	6.00265	0.8306	0.007703	0.05585
neg_3488	Pyrenophorol	up	neg	-	311.1512	6.130417	1.3447	0.008205	0.04866
neg_3787	Cerasidin	down	neg	-	389.1257	4.676667	0.7302	0.04122	0.1369
pos_3690	L-Valine, N-(2-hydroxy-3-butenyl)-	down	pos	-	229.154	0.983783	0.8396	0.005477	0.04592
neg_4267	Deoxyuridine	up	neg	951-78-0	273.0736	1.715067	1.2777	0.01188	0.06246
neg_3431	Setarin	up	neg	31005-07-9	201.0557	6.211033	1.1774	6.03E-08	5.78E-06
neg_329	Citrulline	down	neg	372-75-8	174.0881	0.57875	0.8338	0.000153	0.003263
pos_3149	Morin	up	pos	480-16-0	303.0488	5.047617	1.188	0.006654	0.05205
pos_2917	Macrophorin B	down	pos	85764-12-1	416.2452	6.0748	0.8385	0.004487	0.04074
neg_1752	(Z)-[(4-hydroxyphenyl)acetaldehyde oxime]	down	neg	-	347.126	3.568017	0.7262	0.03851	0.1302
pos_1190	PC(15:0/20:4(8Z,11Z,14Z,17Z)-2OH(5S,6R))	up	pos	-	422.7539	2.999983	1.224	0.03557	0.1373
neg_2465	N-Stearoyl Histidine	up	neg	-	466.3322	7.19125	1.1366	0.000203	0.004034
pos_993	Cytidine 2'-phosphate	down	pos	85-94-9	288.0387	1.694517	0.8315	0.01104	0.06791
neg_579	PE(P-18:0/22:4(7Z,10Z,13Z,16Z))	up	neg	-	800.5503	6.442933	1.1724	0.001786	0.0176
neg_4385	Trans-Resveratrol 3,4'-disulfate	up	neg	-	424.944	1.351483	1.3085	0.01237	0.0638
neg_513	Xi-8-Hydroxyhexadecanedioic acid	down	neg	-	301.2029	6.0984	0.8316	0.008741	0.05068
neg_1993	Parylene C	down	neg	-	321.045	5.76505	0.8086	0.004679	0.03373
neg_3708	Quercitrin	up	neg	522-12-3	447.0952	5.385917	1.2389	0.02468	0.09947
neg_1804	4-Methylbenzoic Acid	up	neg	99-94-5	135.0444	3.97945	1.2351	0.002892	0.0243
pos_2170	Ixocarpalactone A	up	pos	71801-45-1	1031.527	7.776367	1.1391	0.001905	0.02619
pos_222	1,1'-(Tetrahydro-6a-hydroxy-2,3a,5-trimethylfuro[2,3-d]-1,3-dioxole-2,5-diyl)bis-ethanone	down	pos	18114-49-3	276.1433	1.694517	0.8954	0.000344	0.008098
pos_1744	Ethisterone	up	pos	434-03-7	330.245	6.79685	1.1687	0.03438	0.1345
neg_509	Sorbitan laurate	down	neg	1338-39-2	345.2294	6.057767	0.8467	0.008166	0.0485
neg_37	13(S)-Hydroperoxylinolenic acid	down	neg	67597-26-6	309.2081	6.331033	0.8752	0.000341	0.005914

ID	Metabolite	Regulate	Mode	CAS ID	M/Z	Retention time	FC(RF/RC)	P_value	FDR
neg_4497	Cytidine monophosphate	down	neg	63-37-6;84-52-6	322.0469	1.048283	0.8311	0.006663	0.04226
neg_3378	9,10-DHOME	down	neg	125356-86-7	313.2394	6.2912	0.8879	0.000164	0.00342
pos_3404	5-Chloro-2'-deoxyuridine	up	pos	-	262.0371	2.709517	1.1548	0.001241	0.02029
pos_3450	N6-Acetyl-5S-hydroxy-L-lysine	up	pos	-	472.2389	2.583167	1.1159	0.003196	0.03322
neg_4633	3-dehydroshikimate	up	neg	-	401.0709	0.840767	1.1186	3.20E-06	0.000152
pos_73	1-Benzyl-1,2,3,4-tetrahydroisoquinoline	up	pos	19716-56-4	488.3097	7.099583	1.0966	0.00095	0.01703
neg_4845	Dihydroxyphenyl-valerolactone	down	neg	-	229.0485	0.642133	0.8366	0.006215	0.04057
neg_2381	N-Linoleoyl Phenylalanine	down	neg	-	426.3032	6.8568	0.8413	0.006839	0.04264
pos_3852	METHACHOLINE	up	pos	55-92-5	160.1328	0.657867	1.1383	0.003044	0.03292
neg_3441	Rishitin	down	neg	18178-54-6	267.1609	6.203033	0.7991	0.005752	0.03869
pos_3401	2,4-Thiazolidinedicarboxylic acid, 2-methyl-	up	pos	-	192.032	2.714433	1.1289	0.00021	0.006085
neg_1755	L-Dopa	up	neg	59-92-7	178.0507	3.590883	1.1215	0.000217	0.004233
pos_2488	LysoPA(O-18:0/0:0)	up	pos	-	466.3281	7.006317	1.0937	0.001936	0.02619
pos_1596	9-Hpode	down	pos	29774-12-7	313.2362	6.23715	0.8727	0.004793	0.04205
neg_3392	Dolicholide	down	neg	85228-11-1	523.3296	6.267233	0.8407	0.007382	0.04486
pos_2668	MG(0:0/22:4(7Z,10Z,13Z,16Z)/0:0)	down	pos	-	424.3415	6.548333	0.887	0.004784	0.04205
pos_1115	Milrinone	up	pos	78415-72-2	250.0372	2.683133	1.0963	0.000103	0.0044
neg_3690	Empagliflozin	down	neg	-	487.0904	5.541767	0.8184	0.03032	0.1118
pos_1546	Methyl Jasmonate	down	pos	95722-42-2;1211-29-6	225.1478	6.0972	0.8322	0.03785	0.141
pos_588	Lepidiumterpenyl ester	down	pos	255833-57-9	400.3409	6.392833	0.9132	0.000228	0.006141
pos_1241	5-Hydroxyindoleacetate	up	pos	54-16-0	192.0648	3.39885	1.1351	0.01784	0.08939

ID	Metabolite	Regulate	Mode	CAS ID	M/Z	Retention time	FC(RF/RC)	P_value	FDR
pos_3136	4H-Pyrido(1,2-a)pyrimidin-4-one, 9-((4-acetyl-3-hydroxy-2-propylphenoxy)methyl)-3-(1H-tetrazol-5-yl)-	up	pos	-	385.1413	5.2195	1.1342	0.004335	0.03994
pos_3227	Penitrem E	up	pos	78213-66-8	617.3597	3.791933	1.1297	0.04758	0.1647
pos_2982	(8R,9S,10S,13S,14S,17R)-16-Fluoro-17-hydroxy-10,13-dimethyl-1,2,4,5,6,7,8,9,11,12,14,15,16,17-tetradecahydrocyclopenta[a]phenanthren-3-one	up	pos	-	331.2041	5.9364	1.1005	0.000241	0.006294
neg_2311	Hexadecanedioic acid	down	neg	42150-38-9;505-54-4	285.2079	6.626233	0.8709	0.001307	0.01454
neg_3249	2-(4-Hydroxy-3,5-di-tert-butylphenylthio)-hexanoic acid	up	neg	-	397.2034	6.56245	1.1377	0.000101	0.002394
pos_2957	20-Hydroxy-leukotriene E4	down	pos	111844-33-8	473.2653	5.989617	0.8819	0.02007	0.09512
neg_4032	Salicyluric acid	up	neg	487-54-7	194.0458	3.00685	1.1849	0.001383	0.015
neg_1964	Phellopterin	up	neg	2543-94-4	345.0994	5.5628	1.2134	0.0152	0.07364
pos_2950	22-Hydroxydocosanoic acid	down	pos	-	395.2892	5.994867	0.8796	0.01521	0.08155
neg_2122	9,10,13-TriHOME	down	neg	29907-57-1	329.2343	6.1464	0.9077	4.02E-05	0.001184
neg_2	Deoxycholic acid	down	neg	83-44-3	391.2867	6.394917	0.9029	0.01688	0.07804
pos_3117	Asteltoxin	up	pos	79663-49-3	457.1622	5.5034	1.0973	0.00015	0.005302
neg_3593	Menthone 1,3-glyceryl ketal	down	neg	63187-41-7	273.1716	5.97855	0.8378	0.004825	0.03449
pos_3119	Cajaisoflavone	up	pos	72578-99-5	514.1838	5.4553	1.1207	0.0141	0.07831
neg_4300	Trifluoroquinolone	up	neg	-	443.0445	1.664067	1.1564	0.00098	0.01221
pos_2864	Jasmonic acid	up	pos	59366-47-1;6894-38-8	193.1218	6.175017	1.1227	0.003187	0.03322
neg_523	9-F1-phytoprostane	down	neg	-	327.2187	6.122433	0.9005	0.000513	0.008133

**ID**	**Metabolite**	**Regulate**	**Mode**	**CAS ID**	**M/Z**	**Retention time**	**FC(RF/RC)**	**P_value**	**FDR**
neg_3346	Auberganol	down	neg	102490-02-8	285.2079	6.347	0.88	0.001686	0.01718
neg_3288	Lysopc(18:3)	up	neg	-	562.3176	6.450933	1.0934	0.000787	0.01098
pos_1860	5-androstene-3,17-dione	up	pos	571-36-8	590.4228	7.426317	1.0871	0.000194	0.005773
neg_1912	Scopoletin	down	neg	92-61-5	237.0409	5.121717	0.8107	0.02441	0.09884
neg_3289	(-)-Usnic acid	up	neg	6159-66-6	343.0833	6.450933	1.1401	0.001828	0.01788
neg_4130	Cystathionine ketimine	up	neg	87254-95-3	248.024	2.68165	1.0937	2.45E-05	0.000776
pos_3850	S-(2-Carboxyethyl)-L-cysteine	up	pos	-	194.0475	0.657867	1.1285	0.03298	0.1309
neg_3672	5-Formiminotetrahydrofolic acid	down	neg	2311-81-1	493.1527	5.6735	0.8393	0.02597	0.1021
neg_1931	Equol 7-O-glucuronide	down	neg	-	417.1209	5.2696	0.878	0.002885	0.02428
neg_1922	Tricin 7-O-Glucuronide	down	neg	-	505.1014	5.188667	0.8467	0.01562	0.07476
neg_431	Solerol	up	neg	27610-27-1	191.0559	0.634233	1.1094	0.00082	0.01123
pos_1385	Hydrocodone	up	pos	125-29-1	317.188	5.7566	1.1056	0.001753	0.02519
pos_1223	Paucine	down	pos	29554-26-5	251.1381	3.235483	0.8744	0.02712	0.1163
neg_1457	Urea aspartate	down	neg	-	170.0204	1.721067	0.8678	0.001253	0.01424
pos_3696	Thiomorpholine 3-carboxylate	down	pos	20960-92-3	148.0424	0.9682	0.9242	0.002752	0.0312
pos_1651	LysoPC(18:3(6Z,9Z,12Z)/0:0)	up	pos	-	540.3044	6.462767	1.0714	0.002277	0.02819
pos_963	Valylhydroxyproline	up	pos	90965-79-0	263.1592	1.501183	1.109	0.005268	0.04515
pos_3428	6-Thioguanosine	up	pos	-	282.0633	2.649733	1.0933	0.004349	0.03994
neg_1733	Ethyl 3-aminobenzoate	up	neg	-	146.0606	3.4043	1.2314	0.03102	0.1132
neg_3398	Arenobufagin	up	neg	464-74-4	397.2035	6.2589	1.1042	4.10E-05	0.001201
pos_3663	Coniferin	up	pos	531-29-3;124151-33-3	406.1441	1.053833	1.1105	0.01226	0.07206
pos_3152	Karanjin	up	pos	-	310.1096	4.97495	1.0817	0.000184	0.005662

ID	Metabolite	Regulate	Mode	CAS ID	M/Z	Retention time	FC(RF/RC)	P_value	FDR
neg_3095	7-[(1R,2R,3R)-3-Hydroxy-2-[(1E,3S)-3-Hydroxyoct-1-en-1-yl]-5-oxocyclopentyl]heptanoylcarnitine	up	neg	-	532.3044	6.991967	1.1187	0.001517	0.01607
neg_4129	Threonylhydroxyproline	up	neg	844641-04-9	267.0742	2.68165	1.1093	0.001538	0.01613
pos_770	L-Canaline	down	pos	496-93-5	176.1024	0.596333	0.9192	0.001526	0.02354
neg_3412	Tetradecanedioic acid	down	neg	821-38-5	257.1764	6.23495	0.8841	0.00228	0.0209
pos_2471	Isoetharine	down	pos	530-08-5	542.3201	7.099583	0.9464	0.003417	0.03441
pos_1673	Cycloheximide	up	pos	66-81-9	246.1514	6.5561	1.094	0.007603	0.05564
pos_3628	2-Mercapto-3-furan-2-ylpropenoic acid	up	pos	-	188.037	1.194117	1.0865	0.001748	0.02519
neg_2135	Pisatin	up	neg	469-01-2	359.0786	6.170417	1.1039	0.00017	0.003493
neg_4411	Ethylene carbonate	up	neg	96-49-1	221.0305	1.2638	1.1851	0.01236	0.0638
pos_1062	5-Hydroxy-L-tryptophan	down	pos	4350/9/8	221.0914	2.34465	0.8889	0.03455	0.1348
neg_2347	Ricinoleic acid	down	neg	141-22-0	297.2442	6.73775	0.9294	0.000521	0.008133
neg_2261	PE(22:5/0:0)	up	neg	-	526.2964	6.47475	1.1279	0.02922	0.1093
neg_1529	Serylalanine	down	neg	6403-17-4	213.0266	2.317217	0.8912	0.00134	0.0148
pos_1225	Indoleacetaldehyde	up	pos	2591-98-2	160.0753	3.259817	1.0981	0.00479	0.04205
pos_3683	N2-Acetylornithine	down	pos	6205/8/9	175.1072	1.0071	0.9204	0.002211	0.02781
neg_1427	Mevalonic acid	down	neg	150-97-0;17817-88-8	147.0657	1.6761	0.8825	0.001619	0.0167
neg_3692	Exo,exo-1,8-Epoxy-p-menthane-2,6-diol	down	neg	38223-98-2	231.1241	5.524433	0.8624	0.005707	0.03869
neg_2424	Hyperforin	up	neg	11079-53-1	535.3816	6.99995	1.0867	0.005944	0.0395
pos_430	PE(P-16:0/0:0)	up	pos	-	438.2966	6.72695	1.0682	0.005832	0.04743
pos_2	Ursodeoxycholic acid	down	pos	128-13-2	785.5878	6.40055	0.9544	0.001424	0.02264
neg_1414	3-Methylxanthine	down	neg	1076-22-8	165.0413	1.636283	0.849	0.01537	0.074
neg_3170	LysoPE(18:2(9Z,12Z)/0:0)	down	neg	85046-18-0	476.2801	6.7536	0.9431	0.000271	0.004951
pos_1005	Thymine	up	pos	65-71-4	127.0501	1.725667	1.0817	0.000438	0.009866

ID	Metabolite	Regulate	Mode	CAS ID	M/Z	Retention time	FC(RF/RC)	P_value	FDR
pos_3301	Isovalerylcarnitine	down	pos	31023-24-2	246.1692	3.151417	0.9328	0.01003	0.06557
neg_4474	Pseudouridine 5'-phosphate	down	neg	1157-60-4	305.0207	1.104267	0.9081	0.002189	0.02029
neg_545	Chenodeoxycholylserine	down	neg	-	478.319	6.2589	0.9142	0.0358	0.1244
pos_1519	Anandamide	up	pos	94421-68-8	330.2781	5.994867	1.126	0.04429	0.1566
neg_3231	1-Nonadecanoyl-glycero-3-phosphoserine	up	neg	-	538.3171	6.6023	1.0857	0.000597	0.008847
pos_1731	Tetraethylene glycol monododecyl ether	down	pos	-	385.2917	6.734733	0.9282	0.01015	0.06557
neg_3174	Alpha-Linolenoyl ethanolamide	down	neg	-	366.2656	6.7536	0.9118	0.006268	0.04067
neg_1848	N-Acetyl-L-phenylalanine	down	neg	2018-61-3	206.0823	4.48705	0.8717	0.04083	0.136
pos_3359	2-n-Propylthiazolidine-4-carboxylic acid	up	pos	-	176.0735	2.917283	1.0593	0.000228	0.006141
pos_1297	1-(D-3-Mercapto-2-methyl-1-oxopropyl)-L-proline	up	pos	-	281.0944	4.052083	1.0768	0.003823	0.03707
neg_772	Keto-3-deoxy-D-manno-octulosonic acid	down	neg	-	273.0389	0.769333	0.915	0.004202	0.03164
neg_3076	12-hydroxyicosanoic acid	down	neg	-	327.2914	7.079767	0.9123	0.004786	0.03427
pos_1272	Phenylbutyrylglutamine	up	pos	-	275.1414	3.700867	1.0735	0.01186	0.07076
pos_3602	Beta-Tyrosine	down	pos	-	204.0624	1.392267	0.9417	0.000268	0.006824
pos_1643	Docosahexaenoic acid	down	pos	6217-54-5	361.2725	6.4395	0.9258	0.01158	0.06984
neg_3461	N-Arachidonoyl Proline	down	neg	-	446.2929	6.170417	0.9163	0.02637	0.1029
pos_49	LysoPE(0:0/18:2(9Z,12Z))	down	pos	-	478.2915	6.501683	0.9579	0.002009	0.02652
pos_248	PGP(i-18:0/PGJ2)	up	pos	-	318.4912	2.583167	1.09	0.04839	0.1663
neg_1018	2-Methylthiazolidine-4-carboxylic acid	up	neg	-	192.0334	0.650083	1.1129	0.03994	0.1337
pos_2302	LysoPC(20:2(11Z,14Z)/0:0)	up	pos	-	570.3514	7.6442	1.0786	0.03049	0.1242
pos_1327	Ferreirin	up	pos	32898-79-6	344.115	4.660517	1.0663	0.007186	0.05416
pos_3890	Meso-2,6-Diaminoheptanedioate	up	pos	583-93-7	191.102	0.61165	1.0632	0.000857	0.0161
pos_52	PC(18:2/0:0)	down	pos	-	520.3384	6.6726	0.9638	0.000952	0.01703
neg_565	9(S)-HOTrE	down	neg	-	293.2131	6.370917	0.9235	0.008122	0.04844
neg_3396	Daphniphylline	up	neg	15007-67-7	526.3535	6.2589	1.0962	0.03993	0.1337

ID	Metabolite	Regulate	Mode	CAS ID	M/Z	Retention time	FC(RF/RC)	P_value	FDR
pos_1055	Oxolinic acid	up	pos	14698-29-4	279.1	2.2796	1.0669	0.01897	0.09224
neg_3345	(R)-1-Octen-3-ol	down	neg	-	315.2549	6.347	0.9194	0.006132	0.04028
neg_3527	Formononetin	down	neg	485-72-3	267.067	6.08175	0.8758	0.04284	0.1398
neg_2439	AVOCADENE ACETATE	down	neg	-	327.2551	7.087717	0.922	0.000909	0.01182
neg_2397	Vaccenic Acid	down	neg	506-17-2;693-72-1	327.2551	6.928417	0.9099	0.005946	0.0395
neg_3459	ROSAMICIN	down	neg	-	580.3516	6.170417	0.9218	0.02438	0.09884
pos_276	2-Oxo-4-methylthiobutanoic acid	down	pos	583-92-6	166.0528	0.604017	0.9492	0.003146	0.03322
neg_3822	Protocatechuic Acid	down	neg	99-50-3	153.0188	4.323467	0.9073	0.01004	0.05569
neg_3783	N-Acetyl-D-phenylalanine	down	neg	10172-89-1	206.0823	4.7133	0.9323	0.000261	0.004813
neg_3195	Nevadensin	up	neg	10176-66-6	343.0833	6.68995	1.0867	0.008818	0.05094
pos_2745	Ligusticide	up	pos	-	381.2048	6.4161	1.0564	0.00122	0.02012
pos_3212	Azacosterol	up	pos	-	406.3778	3.9353	1.061	0.002382	0.02865
neg_2891	PC(18:3(6Z,9Z,12Z)/16:0)	up	neg	-	800.5495	7.646817	1.1069	0.0379	0.129
pos_2973	Ethofumesate	up	pos	26225-79-6	328.1201	5.958417	1.0544	0.007065	0.05345
neg_2421	LysoPE(P-18:0/0:0)	up	neg	174062-73-8	464.3162	6.991967	1.0658	0.01314	0.06653
pos_58	PC(18:1(6Z)/0:0)	down	pos	-	544.3364	7.099583	0.9542	0.01833	0.09054
neg_1914	9-(2,3-dihydroxypropoxy)-9-Oxononanoic Acid	down	neg	-	261.135	5.1304	0.8991	0.01903	0.08422
pos_3677	Vigabatrin	down	pos	60643-86-9	174.0504	1.022667	0.9439	0.01491	0.08107
pos_1171	5'-Methylthioadenosine	up	pos	2457-80-9	298.0959	2.939067	1.0511	0.008614	0.06002
neg_4932	2-Hydroxy-L-methionine	down	neg	-	164.0383	0.586717	0.9327	0.000981	0.01221
pos_1251	Methionyl-Proline	up	pos	-	247.1102	3.470883	1.0728	0.03786	0.141
pos_3184	ARGININOSUCCINATE	up	pos	2387-71-5	329.0887	4.457167	1.0515	0.006924	0.05328
neg_4445	4'-Azidocytidine	up	neg	-	319.0552	1.184133	1.0563	0.000127	0.002806
pos_85	Deoxycholylserine	down	pos	-	480.3311	6.252733	0.9432	0.04851	0.1664

ID	Metabolite	Regulate	Mode	CAS ID	M/Z	Retention time	FC(RF/RC)	P_value	FDR
pos_573	Alpha-Eleostearic acid	down	pos	506-23-0	296.2574	6.4317	0.9568	0.004866	0.04257
neg_1852	Acetyl-DL-Leucine	down	neg	1188-21-2	172.0976	4.4983	0.9424	0.000199	0.00397
neg_3969	3-(4-Hydroxyphenyl)lactate	down	neg	306-23-0	181.0504	3.163567	0.9235	0.005405	0.03745
neg_584	PE(18:2/0:0)	down	neg	-	476.2799	6.490667	0.9528	0.003745	0.02919
pos_838	Erythro-4-hydroxy-L-glutamate(1-)	up	pos	-	205.0812	0.820533	1.0487	0.007245	0.05416
neg_1653	2-Methylglutaric Acid	down	neg	617-62-9	145.0501	2.999217	0.9436	7.08E-05	0.00183
pos_2645	4-Trimethylammoniobutanoic acid	down	pos	407-64-7	184.0726	6.602733	0.9583	0.003726	0.03655
pos_2522	Polygonal	up	pos	-	508.3384	6.889867	1.0578	0.03169	0.1273
neg_2359	2-Hydroxystearic acid	down	neg	26531-80-6	299.2599	6.761583	0.9457	0.007926	0.04741
pos_432	PE(17:0/0:0)	up	pos	-	468.307	6.734733	1.0426	0.01022	0.06557
pos_40	Gamma-D-Glutamylglycine	up	pos	-	205.0812	0.61165	1.0504	0.0171	0.08713
pos_1104	Cotinine	up	pos	486-56-6	209.1277	2.664867	1.0637	0.007419	0.05494
neg_1009	Glyceric Acid	down	neg	473-81-4	105.0184	0.642133	0.9457	0.002426	0.02172
pos_580	SM(d18:0/20:4(6E,8Z,11Z,13E)-2OH(5S,15S))	down	pos	-	807.5708	6.40055	0.96	0.01417	0.07844
pos_3538	Pro-Ile	down	pos	-	229.1539	1.725667	0.9352	0.03713	0.1398
pos_1931	LysoPC(20:5(5Z,8Z,11Z,14Z,17Z)/0:0)	down	pos	-	542.3201	7.652	0.9616	0.01777	0.08934
neg_629	13-HODE	down	neg	29623-28-7	295.2286	6.832933	0.9568	0.004117	0.03134
pos_439	24,24-Difluoro-1alpha,25-dihydroxyvitamin D3	up	pos	-	435.3067	6.835567	1.0542	0.02403	0.1071
neg_4836	5-Diazouracil	down	neg	2435-76-9	183.0157	0.650083	0.9427	0.000479	0.007761
neg_1731	2-Hydroxyquinoline	up	neg	70254-42-1	190.0507	3.39715	1.0807	0.0161	0.07596
neg_4180	7,4'-Dihydroxy-8-methylflavan	up	neg	75412-98-5	277.0871	2.317217	1.0679	0.02309	0.09601
